# RNA epigenetic modifications as dynamic biomarkers in cancer: from mechanisms to clinical translation

**DOI:** 10.1186/s40364-025-00794-y

**Published:** 2025-06-07

**Authors:** Yingchao Zhao, Xiang Chen, Xingli Zhang, Hong Liu

**Affiliations:** 1 Department of Dermatology, Xiangya Hospital, Central South University, Changsha, Hunan China; 2 National Engineering Research Center of Personalized Diagnostic and Therapeutic Technology, Changsha, Hunan China; 3 Hunan Key Laboratory of Skin Cancer and Psoriasis, Changsha, Hunan China; 4 Hunan Engineering Research Center of Skin Health and Disease, Changsha, Hunan China

**Keywords:** Anti-tumor therapy, Cancer, Epitranscriptomics, RNA modification

## Abstract

RNA modifications are crucial for post-transcriptional gene regulation. Research on RNA modifications has become a novel frontier of epitranscriptomics. Up to now, over 170 kinds of modifications have been identified on mRNA and diverse non-coding RNA. Three classes of proteins (writers, erasers, and readers) regulate the addition, removal, and identification of epigenetic marks, thus affecting RNA biological functions. Increasing evidence identifies the dysregulation of RNA modifications in different cancer types and the therapeutic potential of targeting RNA-modifying enzymes. The ability of RNA modifications to improve mRNA stability and translation efficacy and decrease immunogenicity has been exploited for the clinical use of mRNA cancer vaccines. This review aims to shed light on several vital cap, tail, and internal modifications of RNA with a focus on the connection between RNA epigenetic pathways and cancer pathogenesis. We further explore the clinical potential of RNA modifications as dynamic biomarkers for cancer diagnosis, prognosis, and therapeutic response prediction, addressing both technological challenges and translational opportunities. Finally, we analyze the limitations of current studies and discuss the research focus in the future.

## Introduction

Epigenetic modification refers to heritable changes in gene function without any changes in nucleotide sequence [1]. This modification includes the regulation of selective transcriptional expression of genes (DNA methylation, histone modifications, genomic imprinting, chromatin remodeling) and post-transcriptional regulation of genes. Extensive research on chemical modifications of DNA and histone highlighted their effect on biological processes [2, 3]such as growth, aging, and human diseases. Likewise, RNA post-transcriptional modifications are widespread in cells and provide additional layers of information and regulation for the sequence and structure of the transcripts.

The first RNA nucleoside modification was discovered in the 1950s [4]. Thus far, up to 170 RNA modifications have been identified on four kinds of RNA bases (A, U, C, G) and ribose in any known RNA species [5]. As an intermediate in protein synthesis, messenger RNA (mRNA) contains a 5’cap and the poly (A) tail at the 3’end to facilitate pre-mRNA splicing, polyadenylation, nuclear export, mRNA stability, translation initiation, and recycling [6]. It follows that modifications affecting 5’cap or poly (A) tail could influence biological functions. For instance, alternative polyadenylation (APA) leads to numerous transcripts with differing 3’ends and participates in regulation of RNA metabolism [7]. After the discovery of the cap and tail modifications, internal RNA modifications were found and investigated. The most common mRNA modifications include N6-methyladenosine (m6 A), N1-methyladenosine (m1 A), adenosine-to-inosine editing (A-I), 5-methylcytosine (m5 C), 5-hydroxymethylcytosine (hm5 C), pseudouridylation (Ψ), and ribose-methylation (2’-O-Me) [8].

As effector molecules, transfer RNA (tRNA) and ribosomal RNA (rRNA) are the most heavily modified. On average, eukaryotic tRNAs contain 13 modifications per molecule, which contributes to the fidelity and efficiency of decoding, folding, localization, and cellular stability [6]. In addition to tRNA and rRNA, the latest realization that non-coding RNAs (ncRNAs) have a direct influence on gene expression brings the modifications of other ncRNAs, such as microRNA (miRNA), long ncRNA (lncRNA), and circular RNA (circRNA) into focus [9–11]. Overall, abundant modifications play vital roles in RNA metabolism and regulate various cellular processes. Recent studies indicate that the dysfunction of RNA modification pathways is involved in the pathogenesis of human diseases, benefiting from the development of novel and improved techniques [11–14]. The great progress of next-generation sequencing (NGS), sensitive and high-throughput mass spectrometry leads to the emerging area of RNA epitranscriptomics [15–17].

Epigenetic modifications of RNA are interrelated to cancer, which is the main threat to human health owing to its high incidence, drug resistance, and poor prognosis. Proliferated knowledge of the functions of RNA modifications in cancer raises the therapeutic potential of anticancer drugs targeting RNA epigenetic pathways [9, 18]. Three classes of proteins ‘writers’, ‘erasers’, and ‘readers’ modulate the addition, removal, and identification of epigenetic marks. Specific inhibitors against these RNA enzymes become latent options for intervention in cancer [19]. The combination of these specific inhibitors and chemotherapy, radiotherapy, targeted therapy, and immunotherapy is a potential synergistic strategy since RNA modification can modulate their efficacy. Numerous bioinformatics studies have established a series of prediction models and identified biomarkers, guiding further research [20, 21].

It is worth mentioning that cancer vaccines have played a critical role in tumor immunotherapy. Among them, mRNA vaccines have attracted wide attention as they are safer, more efficient, and easier to produce. However, the application of mRNA vaccines is limited partly due to their innate immunogenicity and poor stability. Recent studies on RNA modification have broken the above bottleneck. The currently marketed COVID-19 vaccines, BNT162b2 and mRNA-1273, are typical examples of the application of RNA modification in mRNA vaccine development. Some clinical trials of mRNA vaccines for cancer treatment have yielded encouraging results.

Despite rapid progress, significant gaps remain in translating RNA epitranscriptomic insights into clinical practice. This review synthesizes current knowledge on RNA modifications—spanning 5’ cap, poly(A) tail, and internal base modifications—and their roles in cancer pathogenesis. We emphasize the mechanistic connections between RNA epigenetic pathways and various tumor characteristics, including tumorigenesis, invasion, metastasis, drug resistance, and metabolic reprogramming. We evaluate the potential of RNA modification as dynamic biomarkers for diagnosis and prognosis, and discuss challenges in developing RNA-targeted therapies. By addressing these aspects, we aim to bridge mechanistic discoveries with translational opportunities, paving the way for RNA epitranscriptomics to reshape precision oncology.

## Cap modifications

### N 7-methylguanosine (m 7G)

**Fig. 1:**
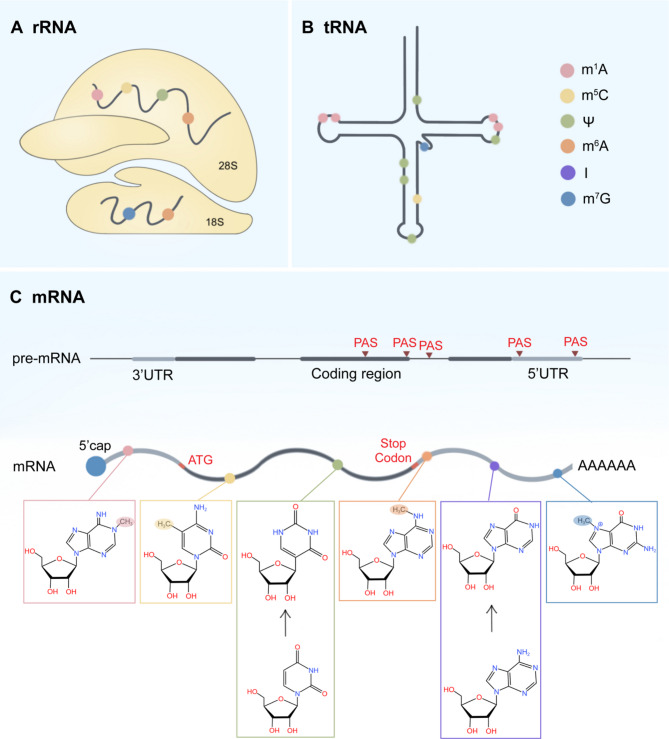
Distribution and chemical structures of RNA modifications. (**A**, **B**) The distribution of internal RNA modifications on rRNA and tRNA. (**C**) The distribution and chemical structure of cap, tail, and internal modifications on pre-mRNA and mRNA. mRNA isoforms arise from APA, which is regulated by the selection of polyadenylation signals (PAS) in proximal, distal, and exonic or intronic regions within the pre-mRNA coding sequence

The m 7G RNA capping is abundant at the 5’end of eukaryotic coding and some noncoding RNAs [22, 23] (Fig. 1). Two enzymes, RNMT (RNA guanine-7 methyltransferase) and RNGTT (RNA guanylyltransferase and 5’phosphatase), finish the three-step process of mRNA capping in humans [24]. Firstly, RNGTT removes the 5’gamma-phosphate of the pre-messenger RNA (pre-mRNA). Subsequently, RNGTT mediates the generation of an atypical 5’-5’ phosphodiester, whereby a guanosine nucleotide is added to the 5’ end of the transcript [25]. The above guanosine is then methylated at position N 7 by RNMT to form m 7G, preventing RNA degradation by 5’exonucleases and affecting RNA processing, export, and translation [26].

Given its important roles in modulation of RNA metabolism, aberrant regulation of m 7G RNA capping has been found in cancer (Table 1). Eukaryotic translation initiation factor 4E (eIF4E), a prooncogenic protein increased in acute myeloid leukemia (AML), specifically binds to m7 G caps and stimulates the export and translation of RNMT and RNGTT [27]. Elevated eIF4E-dependent export of transcripts that encode oncoproteins is related to the condition of patients with AML [28]. Previous studies also confirmed that the over-expression of RNMT facilitates Cyclin D1 (CCND1) translation and promotes mammary epithelial and fibroblast transformation by enhanced m7 G capping of corresponding mRNAs [29]. The reduction of RNMT activity led to decreased proliferation and increased apoptosis in breast cancer cell lines [30]. The aforementioned findings raise the therapeutic potential of antitumor drugs targeting RNA capping enzymes such as RNMT in cancer cells [30].

### Methylation of the 5’-phosphate group of NcRNAs

Non-coding RNAs can carry additional capping modifications, such as the cap methylation of 5’-phosphate groups [31]. The 5’γ-phosphate methylation is mediated by methyl phosphate capping enzyme (MePCE) on 7SK small nuclear RNA (7SK snRNP). MePCE stabilized 7SK which interacts with positive transcription elongation factor b (P-TEFb) complex, providing invasive potential to breast cancer cells [32] (Table 1). Other RNA-methyltransferases, such as BCDIN3 domain-containing protein (BCDIN3D), methylate 5’monophosphate on histidyl tRNA (tRNA^His^) and precursor miRNAs (pre-miRNAs) [33, 34]. BCDIN3D attenuates the expression of miRNAs by inducing 5´-monophosphate dimethylation of the corresponding precursor miRNAs, thereby promoting the invasive potential of breast cancer cells [34].

## Tail modifications

### Alternative cleavage and polyadenylation (APA)

**Fig. 2:**
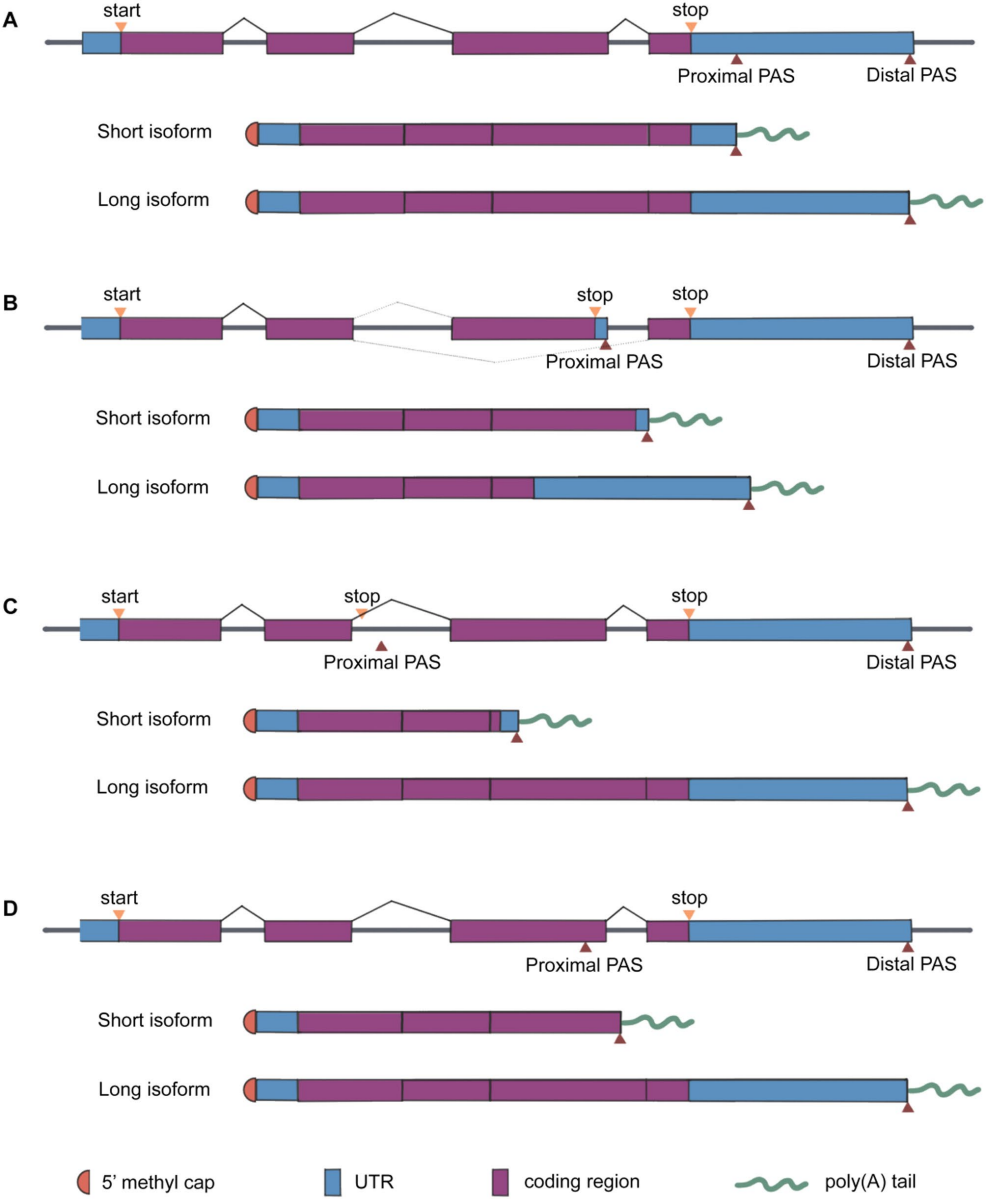
Categories of alternative polyadenylation events. (**A**) Tandem 3’UTR-APA contains two or more PASs in the 3’UTR, leading to 3’UTR length differences between APA isoforms that code for the same protein. (**B**) Alternative terminal exon APA refers to alternative splicing, which results in the altered last exon and therefore the available PAS. (**C**) Intronic APA features a cryptic PAS usage within introns. (**D**) Internal exon APA generates a truncated mRNA isoform that lacks both a 3’UTR and a stop codon via the PAS usage in the upstream exon

The cleavage and polyadenylation (CPA) at the 3’termini of mRNA precursors (pre-mRNA) is essential to mRNA stability and diverse cellular processes, such as gene regulation, mRNA metabolism, protein localization, and diversification [35]. During these processes, the 3’termini of pre-mRNA is cleaved, followed by the addition of adenosine residues to form a poly(A) tail. CPA occurs at polyadenylation sites (PASs) located in introns, internal exons, or 3’ untranslated regions (3’UTRs), generating mRNA transcript isoforms with different coding sequences or 3’UTRs [7]and this phenomenon is termed APA (Fig. 1). The majority of human genes contain multiple alternative PASs. APA in the last exon generates different 3’UTR isoforms which encode the identical gene products, and it is termed tandem 3’UTR APA [36] (Fig. 2). The 3’UTR APA occurring in the proximal PASs usually removes most 3’UTR regulatory elements such as U‑rich elements (AREs), GU‑rich elements (GREs) or PUF protein-binding elements which control mRNA stability, localization, or translation [37]. The other class of APA occurs upstream of the terminal exon, including intronic APA, alternative terminal exon APA, and internal exon APA (Fig. 2). They are all referred to as upstream region APA (UR-APAs) and affect gene regulation [7].

**Table 1:** Functions of RNA epitranscriptomic factors in cancer

Modification type	Regulator	RNA targets	Role	Function	Cancer type
Cap modifications					
m^7^G	RNMT	mRNA	Oncoprotein	Tumor proliferation and apoptosis	Breast cancer
γ-Phosphate methylation	MePCE	7SK snRNP	Oncoprotein	Tumor invasion	Breast cancer
α-Phosphate methylation	BCDIN3D	pre-miRNA	Oncoprotein	Tumor invasion	Breast cancer
Tail modifications					
APA	CPSF1	mRNA	Oncoprotein	Tumor proliferation and migration	HCC
	CPSF4	mRNA	Oncoprotein	Tumor proliferation, migration, invasion, and stemness maintenance	Colon cancer
	NUDT21	mRNA	Tumor suppressor	Tumor proliferation, migration, and invasion	Cervical cancer
	hnRNPC	mRNA	Oncoprotein	Tumor proliferation	Colon cancer
Internal modifications					
m^6^A	METTL3	mRNA	Oncoprotein	Cell differentiation, tumor growth, proliferation, and apoptosis	AML, breast/lung/colon/ prostate cancer
		mRNA	Tumor suppressor	Tumor proliferation	Endometrial carcinoma
	METTL14	pri-miRNA	Tumor suppressor	Tumor growth, metastasis, and invasion	HCC
		mRNA	Oncoprotein	Tumor survival/proliferation, terminal myeloid differentiation	AML
	WTAP	mRNA	Oncoprotein	Tumor proliferation and metastasis	AML, HCC, osteosarcoma
	RBM15	mRNA	Oncoprotein	Tumor proliferation, invasion, migration, and apoptosis	LSCC
	CFL1	seRNA	Oncoprotein	Tumor progression	PDAC
	FTO	mRNA	Oncoprotein	Leukemogenesis, tumor proliferation, and survival	AML
		mRNA	Oncoprotein	Crucial immunotherapy resistance	Cutaneous melanoma
	ALKBH5	mRNA	Oncoprotein	BCSC phenotype, drug resistance	Breast cancer
	YTHDF1	mRNA	Oncoprotein	Tumorigenesis and metastasis	Ovarian cancer
		mRNA	Tumor suppressor	Tumor progression	Ocular melanoma
	YTHDF2	mRNA	Oncoprotein	Tumor progression	Ovarian cancer
	YTHDF3	mRNA	Oncoprotein	Tumor propagation and migration	Ocular melanoma
	YTHDC1	circRNA	Oncoprotein	Tumor proliferation	RMS
	IGF2BP1	mRNA	Oncoprotein	Tumor growth, proliferation, and metastasis	CRC
	IGF2BP3	mRNA	Oncoprotein	Tumor proliferation and apoptosis	B-ALL
m^1^A	TRMT6/ TRMT61A	tRNA	Oncoprotein	Cholesterol synthesis and liver tumorigenesis	HCC
	ALKBH3	tRNA	Oncoprotein	Tumor proliferation, migration, protein synthesis, and drug sensitivity	Various cancer types
		mRNA	Oncoprotein	Tumor invasion	Breast cancer, ovarian cancer
A-to-I editing	ADAR1	mRNA	Oncoprotein	Tumor proliferation	HCC
		miR-25-3p miR-125a-3p	Oncoprotein	Tumor proliferation and drug resistance	Breast cancer
		mRNA	Tumor suppressor	Tumor metastasis	Non-invasive breast cancer
		miR-378a-3p	Tumor suppressor	Tumor malignant progression	Non-metastatic melanoma
	ADAR2	mRNA	Tumor suppressor	Tumor apoptosis	ESCC
		miR-589-3p, miR221,222	Tumor suppressor	Tumor proliferation, invasion, and migration	Glioblastoma
m^5^C	NSUN2	mRNA	Oncoprotein	Tumor proliferation, migration, and invasion	ESCC
		H19 lncRNA	Oncoprotein	Cell differentiation	HCC
	NSUN5	28 S rRNA	Tumor suppressor	Global protein synthesis	Glioma
	NSUN6	tRNA	Tumor suppressor	Tumor proliferation and growth	Pancreatic cancer
Pseudouridine	DKC1	rRNA	Tumor suppressor	Tumor susceptibility of dyskeratosis congenita	Lung and mammary gland tumor, RCC
		lncRNA- PCAT1	Oncoprotein	Tumor proliferation, invasion and apoptosis	NSCLC
	PUS7	tRNA	Oncoprotein	Tumor growth and proliferation	Glioblastoma
m^7^G	METTL1	tRNA	Oncoprotein	Tumor proliferation, migration and invasion	Bladder cancer
		miR-149-3p	Tumor suppressor	Tumor proliferation and chemosensitivity	Colon cancer

Note: HCC, hepatocellular carcinoma; AML, acute myeloid leukemia; LSCC, laryngeal squamous cell carcinoma; PDAC, pancreatic ductal adenocarcinoma; RMS, rhabdomyosarcoma; CRC, colorectal cancer; B-ALL, B-acute lymphoblastic leukemia; ESCC, esophageal squamous cell carcinoma; RCC, renal cell carcinoma; NSCLC, non-small cell lung cancer

APA is modulated by *cis*-regulatory elements in pre-mRNA and many trans-regulators can recognize it. For instance, the cleavage and polyadenylation specificity factors (CPSFs) can identify poly(A) signal (AAUAAA or its variants) [38, 39]. The auxiliary UGUA sequences and GU-/U-rich downstream sequences can be recognized by mammalian cleavage factor I (CFIm) and cleavage stimulation factors (CSTFs), respectively [40–42]. Over 80 APA-related regulators have been recognized within the human pre-mRNA 3’ processing complex till now. Apart from the fundamental elements mentioned above, a number of RNA-binding proteins can also regulate APA, such as retinoblastoma-binding protein 6 (RBBP6), poly A polymerase (PAP) complex, and nuclear polyadenylate-binding protein 1 (PABPN1).

APA mainly interacts with miRNA, RNA-binding protein (RBP), and some APA factors to regulate gene expression and diverse functions [7]. The transcripts with shortened 3’UTR generated by APA can be increased by evading miRNA targeting their 3’UTR [43]. Recent studies discovered that truncated transcripts with shortened 3’UTRs are enriched in tumors, and demonstrated the vital function of 3’UTR shortening in tumor progression, invasion, and prognosis of lung cancer, breast cancer, and gastric cancer [44–46]. Besides, UR-APAs can generate truncated proteins that affect the gene inhibition mechanism in tumors [47–49]. Specifically, the truncated proteins resulting from intronic APA are deficient in the tumor-suppressive features of the homologous full-length proteins, such as FOXN3 and DICER, and some even act as oncogenic factors, including MGA, CARD11, and CHST11 [48].

The dysregulation of APA is also correlated with cancer. On the one hand, the core pre-mRNA 3’end processing complex that regulates the APA formation was found to be abnormally expressed in many cancers. For instance, the up-regulation of cleavage and polyadenylation factor 1 (CPSF1), one of the four subcomplexes of the core pre-mRNA 3’end processing complex, accelerates cell proliferation and migration in hepatocellular carcinoma [50]. CPSF4 can stimulate the growth and progression of colon cancer and lung cancer [51–54]. On the other hand, APA-regulatory proteins, such as Nudix Hydrolase 21 (NUDT21) and heterogeneous nuclear ribonucleoproteins C (hnRNPC), play important roles in cancer [55, 56]. As a tumor suppressor, the knockdown of NUDT21 promotes cervical cancer cell proliferation, invasion, and metastasis [55]. In contrast, hnRNPC is up-regulated and related to clinical outcomes in various cancers [53, 54]. In general, the dysregulation of APA inhibits the expression of tumor suppressor genes and promotes the expression of oncogenes, indicating that APA factors are potential clinical biomarkers and therapeutic targets of cancer.

## Internal modifications

### Adenosine modifications

#### N 6-methyladenosine (m6A)

The methylation of adenosine at position 6 to give m6 A has been best characterized in mRNA [57]. As the most abundant mRNA modification in mammals, m6 A affects the regulation of mRNA splicing, nuclear export, stability, translation efficiency, and subcellular location [58–60]. The m6 A modification sites can be identified at single-nucleotide resolution with the use of Next-Generation Sequencing (NGS) and a modification-specific antibody that precipitates m 6A-modified RNA [61]. These techniques reveal that m6 A modification is enriched in 5’UTRs, near-stop codons, and within coding regions [61]. The m 6A modification also occurs in diverse ncRNAs such as miRNA, rRNA, lncRNA, and circular RNA (circRNA) [62–66] (Fig. 1). Deposition of m 6A is usually enriched in intergenic and intragenic primary miRNAs (pri-miRNAs) which contain typical methyltransferase-like 3 (METTL3) motifs [67]. The m6 A plays a vital role in the processing and maturation of miRNA, thus regulating miRNA-mediated gene silencing [67]. m6 A on rRNA can promote translation initiation and lead to transcript-specific changes [68, 69]. Additionally, m 6A regulates the translation, degradation, and function of the circRNAs through modifying circRNA or changing the methylation state of downstream molecules [70, 71].

**Fig. 3:**
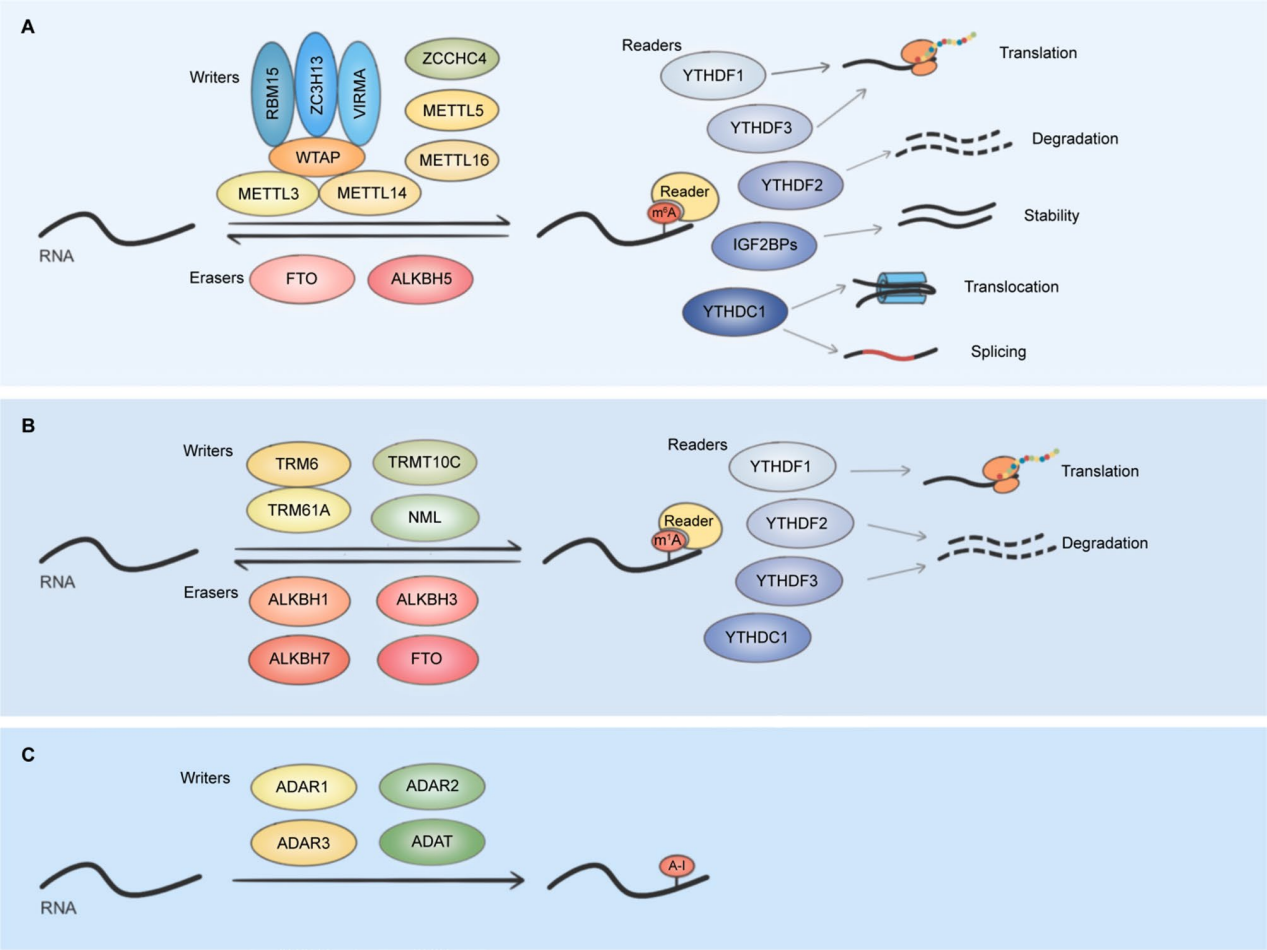
Mechanism of m6 A (**A**), m1 A (**B**), and A-to-I editing (**C**). (**A**) The majority of modifications are regulated by three classes of proteins: writers, erasers, and readers. The METTL3-METTL14-WTAP methyltransferase complex can produce the m6 A modification. RBM15, ZC3H13, and VIRMA are adaptors included in the methyltransferase complex. ALKBH5 and FTO can remove the methyl group in m 6A-modified RNA. YTHDF1-3, IGF2BPs, and YTHDC1 are readers for recognition of m6 A modification. YTHDF1 can facilitate RNA translation while YTHDF2 mainly promotes RNA decay. YTHDF3 exhibits a dual effect relying on its binding partner. IGF2BPs have an influence on the stability of RNA. YTHDC1 affects RNA splicing and the translocation process. **B**) TRMT6 and TRMT61A form an m1 A methyltransferase complex and methylate adenosine in tRNA and mRNA. TRMT10C and NML can catalyze m 1A on tRNA and 28 S rRNA, respectively. The demethylases for m1A modification include ALKBH1, ALKBH3, ALKBH7, and FTO. YTH domain family proteins YTHDF1-3 and YTHDC1 are recognized as m 1A readers. YTHDF1 can boost the translation of m1 A-modified transcripts while YTHDF2 and YTHDF3 reduce the stability of the transcripts. **C)** The adenosine deaminase is regarded as the writer in A-to-I editing, including ADAT family and ADAR family proteins. No research has shown that A-to-I RNA editing is reversible, and no specific RNA binding protein that can recognize this modification has been identified till now

Three classes of proteins, methyltransferases (writers), demethylases (erasers), and binding proteins (readers), modulate the m6 A modification. The METTL3–METTL14–WTAP (Wilms’ tumor 1-associating protein) methyltransferase complex is responsible for the deposition of m 6A on newly synthesized mRNA and miRNA transcripts in the nucleus [72] (Fig. 3). METTL14 acts as the RNA-binding scaffold to recognize the substrate and METTL3 catalyzes the conversion of adenosine to m6 A by its methyltransferase domain [73]. WTAP is a protein with methyltransferase capability (adaptor); it can interact with METTL3 and METTL14 to form a catalytic core on the target RNA [74]. Other adaptors, such as RNA-binding motif protein 15 (RBM15), Vir-like m 6A methyltransferase-associated (VIRMA), and zinc finger CCCH domain containing protein 13 (ZC3H13), are also included in the methyltransferase complex [75]. m 6A modification is also catalyzed by METTL16 which regulates mRNA splicing and stability [76]. In addition, METTL16 can methylate *U6* small nuclear RNA (snRNA), pre-mRNA, and other ncRNAs [76–78].

Two distinct RNA demethylases mainly located in the nucleus, alkB homologue 5 (ALKBH5) and fat mass and obesity-associated protein (FTO), can remove the methyl group in m6 A-modified mRNA [79, 80]. However, recent studies suggest that FTO is more inclined to demethylate N 62-O-dimethyladenosine (m 6A_m_) than m6A [81, 82]. The m6 A modification in mRNA is a dynamic and reversible process regulated by methyltransferases and demethylases (erasers) [83]while the m6 A of rRNA is considered to be constitutive. In eukaryotes, 28 S rRNA is methylated by zinc-finger CCHC domain-containing protein 4 (ZCCHC4), and 18 S rRNA is methylated by METTL5[84, 85]. Most of the m 6 A functions are mediated by readers that can recognize and decode m6 A modification, and then mediate the downstream effect [75]. The best-known m 6A readers are the YTHDF family and IGF2BP family [86]. The YTHDF family is primarily involved in the regulation of pre-mRNA splicing and translation, and the IGF2BP family is responsible for recruiting RNA stabilizers to enhance mRNA stability, affecting tumor progression [87]. Aberrant regulation of m6 A has been identified in many cancers, and it acts as an oncogene or tumor suppressor in different cellular environments.

The role of m 6A methyltransferases in cancer.

METTL3 and METTL14 participate in most m6 A modifications of mRNA and act as tumor suppressors or oncoproteins in various cancers (Table 1). The expression of METTL3 in AML cells is much higher than that in healthy hematopoietic stem/progenitor cells (HSPCs) [88]. CRISPR screens have revealed that *METTL3* is an essential gene for cell growth in AML [89]. In terms of mechanism, METTL3 plays an oncogenic role by methylating the *BCL-2*, *PTEN*, and *c-MYC* mRNAs, facilitating their translation, leading to the inhibition of cell differentiation and apoptosis, thus promoting the progression of AML [88]. For example, oncogene *SP1* can regulate the expression of *c-MYC* and the translation of SP1 depends on the m6 A modification by METTL3 [89, 90]. After the depletion of METTL3, the SP1 protein level declines due to a specific shift of SP1 transcript to a lower molecular weight polyribosome, suggesting less efficient translation and the oncogene *MYC* whose promoter is combined by SP1 is down-regulated as well [89]. METTL3 is also overexpressed in breast, lung, liver, colon, colorectal, and prostate cancer, contributing to the growth, proliferation, and invasion of cancer cells [91–95].

Although not significantly overexpressed, *METTL14* acts as an oncogene by promoting the stability and translation of *MYB* and *MYC* mRNA in AML [96]. METTL16 is necessary for AML cell growth [89]and it plays a vital role in the maturation of lncRNA transfer-associated lung adenocarcinoma transcript 1 (MALAT1), which functions as both a tumor suppressor and an oncogene in different types of cancer [97, 98]. As an adaptor, WTAP is overexpressed in many cancers and serves as an oncogenic protein that can down-regulate the *c-Myc*, *HMBOX1*, and *ETS1* mRNAs in an m6 A-mediated manner, enhancing the proliferation and metastasis of AML, osteosarcoma, and hepatocellular carcinoma (HCC) [99–102]. In addition, RBM15 is significantly increased in laryngeal squamous cell carcinoma (LSCC) [103]. RBM15 improves the stability of transmembrane Bax inhibitor motif-containing 6 (TMBIM6, an anti-apoptotic protein that can play an oncogenic role) through RBM15-mediated m6 A modification of *TMBIM6* mRNA, accelerating LSCC malignant progression [103]. A recent study demonstrated the cofilin family protein CFL1 as a METTL3 cofactor, helping super-enhancer RNA(seRNA) m 6A methylation formation [104]. CFL1 is overexpressed in pancreatic ductal adenocarcinoma (PDAC) and facilitates oncogene transcription relying on the CFL1-METTL3-seRNA m^6^A-YTHDC2/MLL1 axis.

METTL3 and METTL14 work together as a tumor suppressor in endometrial carcinoma [105]. The METTL14 mutation or down-regulation of METTL3 leads to reductions in m6A modification. These changes increase the expression of positive AKT regulator mTORC2 while reducing the expression of negative AKT regulator PHLPP2, activating the AKT pathway and promoting cell proliferation in endometrial carcinoma [105]. Nevertheless, METTL3 and METTL14 are reported to play opposite roles in HCC [93]. METTL3 is up-regulated in HCC and inhibits the suppressor of cytokine signaling 2 (SOCS2) expression through an m6 A-YTHDF2-dependent mechanism, thus stimulating HCC progression [93, 106]. In contrast, METTL14 is down-regulated in HCC, and METTL14 expression can induce an increase of primary microRNA 126 (pri-miR-126) expression to suppress tumor metastasis [106].

The role of m6A demethylases in cancer.

The maladjustment of m 6A erasers is relevant to diverse cancers. As the first identified m 6A demethylase, FTO has been proven to be an oncogenic factor of leukemia [107]glioblastoma [108]and melanoma [109]. FTO reduces the m 6A levels in mRNA of retinoic acid receptor-α (*RARA*) and ankyrin repeat and SOCS box-containing 2 (*ASB2*) and inhibits their expression, promoting leukemic oncogene-mediated cells transformation and leukemogenesis [107]. Furthermore, FTO can stabilize mRNA transcripts of *MYC* and CCAAT enhancer binding protein alpha (*CEBPA*), increasing the proliferation and survival of cancer cells [110]. As a pro-tumorigenic factor, FTO is up-regulated in melanoma as well. FTO enhances the stability of crucial immunotherapy resistance and pro-tumorigenic melanoma cell-intrinsic genes, including programmed cell death 1 (*PDCD1*), CXC-chemokine receptor 4 (*CXCR4*), and SRY-box 10 (*SOX10*), which facilitates melanoma progression and decreases the response to anti-PD-1 blockade [109]. In summary, FTO plays an oncogenic role in these cancers by regulating the expression of its target genes through m6 A RNA modification and its demethylase activity.

ALKBH5 is the second RNA eraser to be identified, which can oxidatively reverse the m6 A modification [79]. In breast cancer, hypoxia-inducible factor 1α (HIF1α) and HIF-2α stimulate the expression of ALKBH5 which can demethylate *NANOG* mRNA and increase its stability, inducing the breast cancer stem cell (BCSC) phenotype [111]. Moreover, a recent study showed that ALKBH5-mediated m 6A demethylation of *GLUT4* mRNA promoted drug resistance to HER2-targeted therapy [112]representing a potential therapeutic target for HER2-positive breast cancer. ALKBH5 was found aberrantly overexpressed in AML, and exerted tumor-promoting effects through post-transcriptional regulation of crucial targets such as the prognosis-associated oncogene *TACC3*, to promote tumorigenesis and self-renewal of cancer stem cell [113].

The role of m6 A-binding proteins in cancer.

The effects of m 6A modification on cancer cells are mainly regulated by m 6A readers. The YTHDF family, including YTHDF1/2/3, is the most widely studied m6 A reader which works through the YTH domain. YTHDF1 is up-regulated in colorectal cancer and ovarian cancer [114, 115]. YTHDF1 serves an oncogenic role in colorectal cancer (CRC) through binding to m 6A sites of Rho guanine nucleotide exchange factor 2 (*ARHGEF2*) mRNA and promoting the translation of ARHGEF2 [114]. Besides, YTHDF1 can increase p65 translation to facilitate myeloid-derived suppressor cell (MDSC) migration, thus resulting in anti-PD-1 resistance and CRC progression [116]. YTHDF1 can also enhance the translation of EIF3C and augment overall translational output concomitantly, leading to tumorigenesis and metastasis of ovarian cancer [115]. In contrast, YTHDF1 has an anti-tumor effect on ocular melanoma by promoting the translation of m 6A-modified mRNA of HINT2 which is a tumor suppressor [117].

YTHDF1 can facilitate RNA translation while YTHDF2 mainly promotes RNA decay [118]. Similar to YTHDF1, YTHDF2 plays an oncogenic role in ovarian cancer. FBW7 can induce proteasomal degradation of YTHDF2 and suppress the YTHDF2-mediated *BMF* mRNA decay, thus inhibiting the progression of ovarian cancer [119]. Moreover, miR-145 can directly target and repress YTHDF2 in ovarian cancer cells [120]. Additionally, YTHDF2 acts as an oncogenic factor in ocular melanoma. Histone acetylation increases the expression of YTHDF2 in ocular melanoma, which stimulates tumorigenesis through degrading m6 A-modified *TP53* and *PER1* mRNAs [121]. YTHDF3 exhibits a dual effect relying on its binding partner [118]. For example, YTHDF3 can recognize *GAS5* mRNA and enhance its degradation in an m 6A-dependent manner [122]. YTHDF3 can also promote *CTNNB1* mRNA translation and potentiates tumorigenicity in ocular melanoma [123].

IGF2BP1, IGF2BP2, and IGF2BP3 are members of the IGF2BP family that specifically recognize m 6A-modified RNA by KH domains and are all highly expressed in carcinogenesis [124]. In CRC, IGF2BP1 promotes tumor growth and cell proliferation by reducing the expression of *E-cadherin* and other epithelial markers [125]. In metastatic melanoma, IGF2BP1 is over-expressed and confers resistance to chemotherapeutic agents [126]. The depletion of IGF2BP1 reduces melanoma metastasis and enhances the sensitivity of melanoma to targeted therapy [126, 127]. IGF2BP3 is up-regulated in mixed lineage leukemia-rearranged B-acute lymphoblastic leukemia (B-ALL) and regulates the stabilization of *MYC* and *CDK6* mRNAs, associated with poor prognosis [128]. A recent study found that m6 A reader YTHDC1 could enhance the production of circRNAs in rhabdomyosarcoma (RMS), promoting RMS proliferation [129]. In conclusion, exploring the targets and their regulation mechanism is the key to understanding the function of m 6A-binding proteins (RBPs) and developing potential anti-tumor drugs.

#### N1-Methyladenosine (m 1A)

m 1A is a reversible methylation modification presenting at the first nitrogen atom of adenosine in tRNA [130]rRNA [131]mRNA [132]and lncRNA [133] (Fig. 1). Among them, m1 A modification mainly occurs at positions 58, 9, and 14 of eukaryotic tRNAs [134]. In mammals, m1A is identified in 28 S rRNA [131]. However, conclusions of the number and location of m 1A modification on mRNAs are contradictory, principally due to the insufficient specificity of the m1 A antibody, calling for further investigation [135–137]. Under physiological conditions, the methyl group of m 1A carries a positive charge which can destroy Watson-Crick base-pairing, thus affecting the processing, translation, structure, and stability of RNAs [134].

As with m6 A, m 1A is regulated by writers, erasers, and readers, and it is aberrantly modulated in cancer. tRNA methyltransferase 6 (TRMT6) and tRNA methyltransferase 61 A (TRMT61A) form an m 1A methyltransferase complex and methylate adenosine in tRNA [138]. TRMT6/TRMT61A complex can also catalyze m1 A on mRNA [135, 139] (Fig. 3). tRNA methyltransferase 10 C (TRMT10C) is another identified writer in tRNA [140]. Moreover, nucleomethylin (NML) plays a vital role in the m1 A methylation of 28 S rRNA [131]. TRMT6/TRMT61A complex is highly expressed in HCC, and it can increase the m 1A modification in a subset of tRNA to elevate peroxisome proliferator-activated receptor-δ (PPARδ) translation, thus stimulating cholesterol synthesis and driving liver tumourigenesis [141] (Table 1). In highly aggressive glioblastoma, the expression of TRMT6/TRMT61A mRNA and tRNA^iMet^ is up-regulated, and elevated TRMT6/TRMT61A can modulate the translation of mRNAs which encode proteins serving as oncogenic factors [142]. A recent study found that TRMT61A-mediated tRNA-m1 A modification could promote the synthesis of MYC protein and upregulate programmed death ligand 1 (PD-L1) expression, leading to the progression of head and neck squamous cell carcinoma [143]. The level of m 1A modification is also elevated in tumors treated with oncolytic herpes simplex virus (oHSV), leading to upregulation of reactive PD-L1. This suggests that targeting TRMT61A can cooperate with oHSV to improve the efficacy of tumor immunotherapy.

m1 A in tRNA is reversible by known RNA demethylase ALKBH1, ALKBH3, ALKBH7 and FTO [81, 144–146]and ALKBH3 can remove the methyl group of m1 A modified mRNA as well [132]. In various cell lines, ALKBH3 can promote the proliferation of cancer cells via demethylating tRNAs and generating tRNA-derived small RNAs [145]. Furthermore, the demethylation of m 1A by ALKBH3 increases the stability of colony stimulating factor-1 (CSF-1) mRNA, promoting the invasion of breast cancer and ovarian cancer cells [147]. It was also suggested that ALKBH3 contributes to cell survival in urothelial carcinoma and non-small-cell lung cancer (NSCLC) by regulating the expression of p21 and p27 which are cell cycle arrest proteins [148, 149]. Overall, ALKBH3 plays an oncogenic role in multiple cancers, mainly by regulating the stability and translation of certain key mRNAs.

Several YTH domain family proteins, YTHDF1-3 and YTHDC1, can bind to m1 A in RNA, suggesting that these YTH domain-containing proteins serve as potential m1 A readers in cells [150, 151]. Since these readers have been intensively studied in m6 A, if their functions in m 1A are confirmed, the possibility of wide-ranging crosstalk between m1 A and m6 A will increase. Increasing studies have demonstrated that m1 A plays a vital role in proliferation, invasion, senescence, and cell death in cancer. Targeting the writers and erasers of m1 A modification is a potential therapeutic strategy for cancer treatment. Further mechanism research requires more effective site-specific programmable m1 A tools to edit and map m1 A modification on the transcriptome.

#### Adenosine-to-inosine (A-to-I)

A-to-I RNA editing, which refers to the C6 hydrolytic deamination of adenosine (A) into inosine (I) mediated by adenosine deaminases, is a common RNA modification in humans. Inosine resembles guanosine (G) in structure so that inosine can pair with cytidine (C), resulting in a misreading of genetic information. A-to-I RNA editing occurs on mRNAs and certain ncRNAs, such as pri-miRNA, pre-miRNA, and lncRNA [152] (Fig. 1). Among them, A-to-I RNA editing in the mRNA coding region can change amino acids, affecting the conformation, stability, subcellular localization, and interaction of proteins. The most frequent A-to-I editing target is double-stranded RNA (dsRNA) composed of inverted *Alu* repetitive elements, which is situated within untranslated regions and introns [153]. Moreover, some pre-miRNA undergoes A-to-I editing as well, resulting in the down-regulation of mature miRNA expression and function [153]. The hydrolytic deamination is caused by adenosine deaminase which is also regarded as the writer in A-to-I editing, and it mainly includes ADAT family and ADAR family proteins [154] (Fig. 3). Unlike m 6A and m1 A, no research has shown that A-to-I RNA editing is reversible, and no specific RNA binding protein that can recognize this modification has been identified till now. Therefore, the discussion below focuses on the role of A-to-I editing writers in cancer.

ADAR family proteins, including ADAR1, ADAR2, and ADAR3, are highly conserved during evolution. These three enzymes all consist of a dsRNA binding domain (dsRBD) and a C-terminal catalytic deaminase domain [155]. Recent studies have demonstrated that the function of ADAR family members relies on different types of cancers. ADAR1 can inhibit the generation of dsRNAs and block the activation of interferon response which is responsible for activating innate immunity so that ADAR1 is up-regulated as an oncogenic factor in various cancers to induce immune silencing [156]. The epigenetic editing catalyzed by ADAR1 on mRNA coding regions may result in an amino acid change and serve an oncogenic role in cancer. For example, ADAR1 mediates the A-to-I editing of AZIN1 transcripts and leads to a serine-to-glycine substitution of AZIN1 in HCC [157] (Table 1). The better stability of the edited AZIN1 protein can promote cell proliferation by suppressing the degradation of cyclin D1 and ornithine decarboxylase. In addition, ADAR1 can mediate A-to-I editing of miR-25-3p and miR-125a-3p binding sites in the 3’-UTR of dihydrofolate reductase (DHFR) and up-regulate the expression of DHFR, inducing cell proliferation and drug resistance in breast cancer [158]. ADAR1 can also modulate the A-to-I editing of tumor suppressor miR-200b and impair the ability of miR-200b to inhibit ZEB1, regulating the invasion capacity of thyroid cancer cells [159].

Besides, some research described ADAR1 as a tumor suppressor [160, 161]. The A-to-I mRNA-edited form of GABAA receptor alpha3 (*Gabra3*) was found in non-invasive breast cancers, and edited Gabra3 repressed the activation of the AKT pathway as well as cancer metastasis [160]. Furthermore, A-to-I edited miR-378a-3p was identified only in non-metastatic melanoma but not in metastatic melanoma [161]. The edited form of miR-378a-3p bound to the 3’-UTR of oncogene *PARVA* preferentially and reduced its expression, inhibiting the malignant progression of melanoma.

ADAR2 mainly acts as a tumor suppressor in cancer through A-to-I editing of mRNAs or miRNAs, in contrast to ADAR1. For instance, ADAR2 is down-regulated in esophageal squamous cell carcinoma (ESCC) [162]. ADAR2 can edit the mRNA of insulin-like growth factor binding protein 7 (*IGFBP7*) and change the protease recognition site of matriptase to stabilize IGFBP7 protein, thus stimulating the apoptosis of cancer cells. Additionally, miR-589-3p, a specific miRNA that can serve as an oncogenic factor, is A-to-I edited by ADAR2 in glioblastoma [163]. Edited miR-589-3p can suppress the proliferation, invasion, and migration of cancer cells, and the edited form in glioblastoma is less than that in normal brain. ADAR2 can also edit miR-221 and miR-222 which are p27-targeting miRNAs and up-regulate the cell cycle inhibitors p27, acting as a tumor suppressor in glioblastoma [164].

Different from ADAR1 and ADAR2, ADAR3 cannot mediate A-to-I RNA editing because of its inactive catalytic domain, while it can regulate A-to-I editing by affecting the function of other ADAR family members [165]. In particular, a comparative study reported that ADAR3 could compete with ADAR2 for binding to glutamate receptor ionotropic AMPA 2 (*GRIA2*) mRNA, inhibiting ADAR2-catalyzed A-to-I editing at the Q/R site of *GRIA2* in glioblastoma [166]. Another main category of adenosine deaminase is ADAT family proteins, containing ADAT1, ADAT2, and ADAT3. These enzymes can catalyze A-to-I editing on tRNA [167].

To sum up, the dysregulation of A-to-I RNA editing has a crucial impact on the biological processes in cancers. In the treatment of a variety of malignancies, including gastric cancer and ESCC, A-to-I editing can aid in prognostic assessment and patient stratification [168–170]. Some studies demonstrated that the level of ADAR1 in ESCC and the level of ADAR2 in HCC are correlated with patient survival, highlighting the potential of A-to-I editing as a novel biomarker for guiding treatment strategies [169, 171]. Furthermore, targeting the writers of A-to-I RNA editing, especially ADAR1, is a potential strategy to fight cancers.

### Cytosine modifications

#### 5-Methylcytosine (m 5C)

m5 C refers to the methylation on C5 of RNA cytosine residues, and it is identified on tRNA, rRNA, mRNA, and some other ncRNAs [172] (Fig. 1). The function of m [5]C modification is diverse depending on RNA types. Specifically, m5 C can regulate the structure and stability of tRNA, which enhances the translation accuracy and efficiency [173, 174]. In rRNA, m5C modification is required for ribosome maturation and protein synthesis [175, 176]. The m5 C enrichment is also found in the GC-rich region, 3’UTR, and around the start codon of mRNA [177, 178]playing a vital role in the export, stability, translation efficiency, and biological function of mRNA [13].

**Fig. 4:**
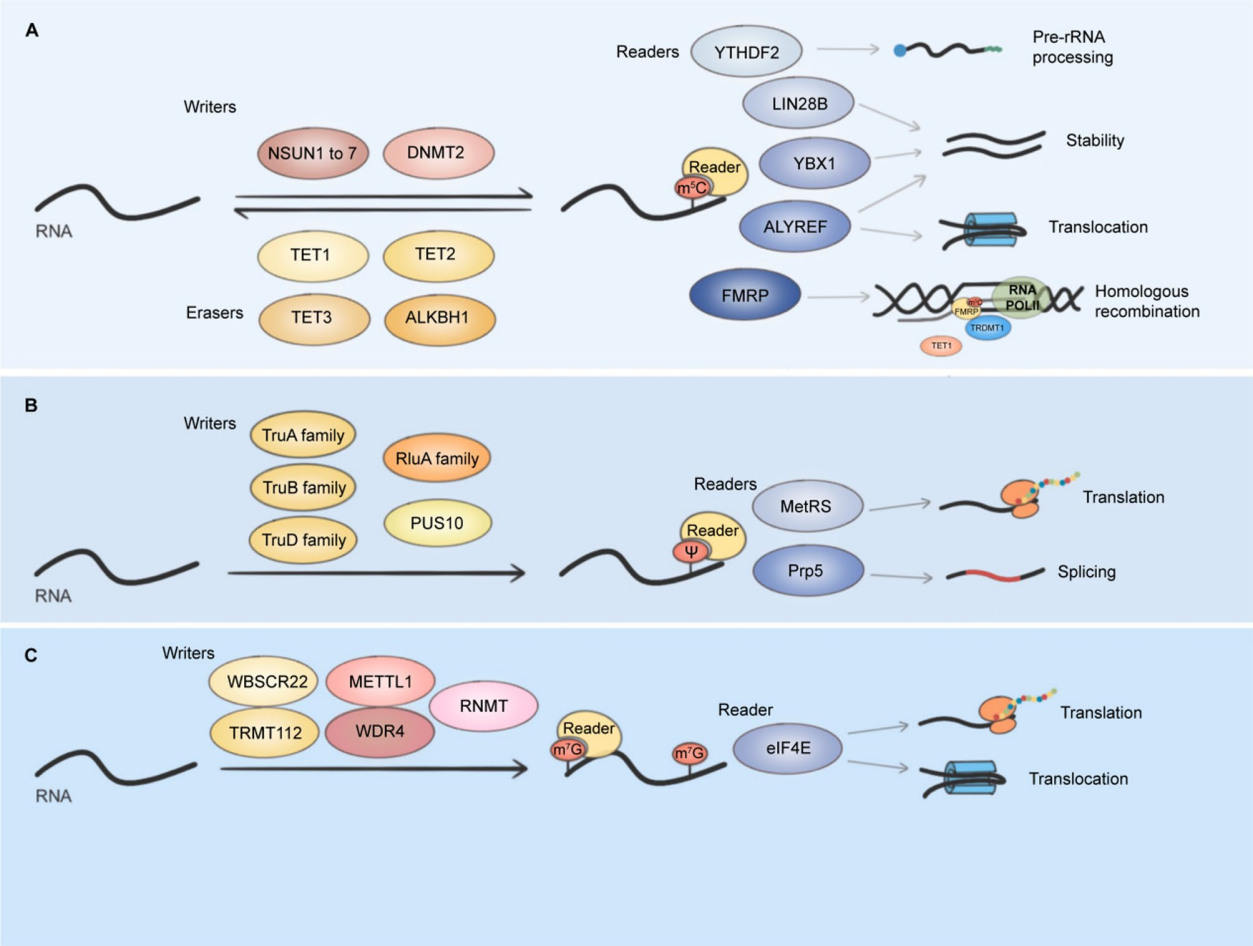
Mechanism of m5 C (**A**), Ψ (**B**), and m7 G (**C**). (**A**) The m5 C writers include NSUN1-7 and DNMT2. TET1-3 and ALKBH1 can remove m5 C modification. Several proteins, such as YTHDF2, LIN28B, YBX1, ALYREF, and FMRP, are identified as m5 C readers. YTHDF2 participates in pre-rRNA processing. LIN28B and YBX1 have similar coldshock domains (CSD), which can recognize m5 C modification and enhance the stability of mRNA. ALYREF can promote RNA translocation and improve RNA stability as well. FMRP recognizes m 5C-modified mRNA and promotes homologous recombination. **B)** The Ψ modification is catalyzed by the TruA family, TruB family, TruD family, RluA family, and PUS10. MetRS can recognize and regulate the translation level of Ψ-modified mRNA and tRNA. Prp5 can bind to Ψ in snRNA, promoting the splicing process of pre-mRNA. **C)** RNMT catalyzes the m 7G capping process at 5’end of transcripts. Internal m 7G is mediated by METTL1/WDR4 complex and WBSCR22/ TRMT112 complex. The reader eIF4E can bind to m7 G caps and stimulate the export and translation of RNMT and RNGTT

Reported m5 C writers include NOL1/NOP2/SUN (NSUN) RNA methyltransferase family which consists of NSUN1 to NSUN7 and DNA methyltransferase-like 2 (DNMT2) [172] (Fig. 4). S-adenosyl-L-methionine (SAM) is used as a methyl donor by these writers to catalyze m5 C modification. NSUN1 and NSUN5 mediate the m5 C modification on 28 S rRNA, stimulating ribosome synthesis and protein synthesis [172, 176]. NSUN2 methylates tRNA, mRNA, and ncRNA vault RNA, regulating the mutation, stability, nuclear export, and translation of RNA [177, 179–181]. NSUN3, NSUN6, and DNMT2 can also modify cytoplasmic tRNA, but with different specificity [12]. NSUN4 catalyzes the m 5C modification on mRNA and the small subunit of mitochondrial rRNA to promote mitochondrial ribosome mutation and translation efficiency [182, 183]. NSUN7 can methylate enhancer RNA (eRNA), regulating cellular energy metabolism [184].

The dysregulation of m5 C writers has been identified in many cancer types. NSUN2 can catalyze the m 5C modification on growth factor receptor-bound protein 2 (*GRB2*) mRNA, stimulating the ESCC progression through Lin-28 homologue B (LIN28B)-dependent GRB2 mRNA stabilization [185] (Table 1). Moreover, aberrant up-regulated NSUN2 is related to poor differentiation of HCC via mediating m5C modification on H19 lncRNA [186]. In contrast with NSUN2, NSUN5 and NSUN6 exhibit tumor-suppressor characteristics in glioma and pancreatic cancer, respectively [187, 188].

Compared with m5 C writers, the studies on erasers and readers are still insufficient. Erasers for m5 C include three members of the ten-eleven translocator (TET) family, TET1-3, and ALKBH1. TET1-3 and ALKBH1 can oxidise m 5C to 5-hydroxymethylcytidine (hm 5C) in mRNA and 5-formylcytidine (f5 C) in tRNA, respectively [189, 190]. In addition, m 5C modification can be recognized by some readers, including Aly/REF export factor (ALYREF), Fragile X mental retardation protein (FMRP), DNA repair protein RAD52, LIN28B, Y-box binding protein 1 (YBX1), and YTHDF2, which affects the stability and nuclear export of mRNA as well as pre-rRNA processing and maturation [175, 177, 185, 191–193]. Some of these erasers and readers have been proven to play a role in cancers, while some of them need further exploration.

## Uridine modification

### Pseudouridine (Ψ)

Pseudouridylation refers to the process in which the pyrimidine ring of uridine (U) rotates 180° with C 3 and C6 as an axis to form pseudouridine (Ψ) which is the most abundant modified nucleoside in human cells [31] (Fig. 1). Normal pyrimidine nucleoside is connected with pentose through an N-C bond which is substituted by a C-C bond in pseudouracil nucleoside. This replacement in Ψ keeps H at the N 1 position, thus the N 1 can serve as a proton donor to generate an additional hydrogen bond and improve RNA stability [194]. Ψ is identified in most classes of RNA with the development of detection methods [195–197]. The Ψ on tRNA can stabilize the structure and coordinate base pairing, and the existence of Ψ on rRNA is essential to ribosome assembly [31]. Ψ is also involved in efficient pre-mRNA splicing and enhancing mRNA stability [198]. Furthermore, pseudouridylation can target the ncRNA telomerase RNA component (*TERC*) and affect telomerase activity [195, 199].

Pseudouridylation is mediated by pseudouridine synthases (PUS) which include TruA family (PUS1, PUSL1, PUS3), TruB family (TRUB1, TRUB2), TruD family (PUS7, PUS7L), RluA family (RPUSD1, RPUSD2, RPUSD3, RPUSD4), and PUS10 in human cells [200] (Fig. 4). Among them, PUS3 and PUS10 catalyze Ψ modification in tRNA, while PUS1, RPUSD2, and RPUSD3 mediate pseudouridylation in mRNA [201–205]. TruB1, TruB2, and PUS7 are pseudouridine synthases for both tRNA and mRNA [203, 205–208]. Moreover, RPUSD4 can catalyze Ψ modification in tRNA, mRNA, and rRNA [203, 205, 209]. Apart from this RNA-independent mechanism catalyzed by PUS, the RNA-dependent approach mediated by a complex that consists of a unique guide RNA component snoRNA/scaRNA and four core proteins, dyskerin pseudouridine synthase 1 (DKC1), NHP2, NOP10, and GAR1 is also confirmed to participate in pseudouridylation on rRNA and snRNA [210, 211]. Although RNA ATPase Prp5 and methionine aminoacyl tRNA^Met^ synthetase (MetRS) have been reported as readers in yeast, no reader has been proven in human cells [212, 213]. In addition, no study indicates that pseudouridylation is a reversible process till now.

The aberrant expression level of Ψ modification and its regulators can influence tumorigenesis. DKC1 is a member of the TruB family, and it can catalyze U-to-Ψ isomerization with a guide RNA and other proteins (NHP2/NOP10/GAR1). The dysregulation of DKC1 has been mostly identified affecting various cancer cells in two opposing ways. Through one mechanism, inactivating mutations of *DKC1* in dyskeratosis congenital cause the lack of pseudouridylation of rRNA, thus leading to dysfunctional translation and cancer progression [214, 215] (Table 1). In another mechanism, the DKC1 is up-regulated and plays an oncogenic role in many cancer types. For instance, DKC1 binds to ribosomal proteins and promotes their expression to promote cancer progression in CRC [216]. In NSCLC, DKC1 synergizes with PCAT1 to stimulate cell proliferation and invasion as well as inhibit apoptosis via the VEGF/AKT/Bcl2/Caspase9 pathway [217]. Additionally, PUS7 can enhance cell growth and proliferation in glioblastoma [218]. Some bioinformatics analyses indicate the functions of PUSs in cancers [219]while the specific mechanisms require further studies.

## Guanosine modification

### Internal 7-methylguanosine (m7 G)

Methylation of guanine at position 7 to give m7 G has been characterized in 5’cap of mRNA and pre-tRNA [220, 221]and internally in tRNA [222]rRNA [223]miRNA [224]and mRNA [225] (Fig. 1). m7 G modification adds a positive charge to the N atom, thereby affecting RNA structure through spatial and electrostatic effects [226]. METTL1/WDR4 complex catalyzes the m7 G modification in tRNA [227]miRNA [224]and mRNA [225, 228]which promotes tRNA expression, miRNA biosynthesis, and mRNA translation (Fig. 4). Another m 7G writer, Williams-Beuren syndrome chromosomal region 22 protein (WBSCR22), can methylate 18 S rRNA in human cells [229]. WBSCR22 cooperates with its metabolic stabilizer TRMT112 to affect the nuclear export of ribosomal subunits and the processing of pre-rRNA [229, 230]. However, whether these functions depend on the m7 G-modifying activity of the WBSCR22/ TRMT112 complex needs further research.

m 7G modification and its regulators can affect tumorigenesis by regulating translation and miRNA processing. Particularly, METTL1 acts as an oncogenic factor in bladder cancer via m 7G modifying certain tRNAs and regulating the expression of epidermal growth factor receptor (EGFR) and EFEMP1 [231] (Table 1). METTL1/WDR4 complex can elevate m7 G modification level in tRNA through the codon-dependent manner decoded by m 7G modified tRNA, enhancing the translation of certain oncogenes such as cyclin D1/D3/E1/A2, *EGFR*, and vascular endothelial growth factor A (*VEGFA*) and promoting the progression of head and neck squamous cell carcinoma (HNSCC), lung cancer and HCC [232–235]. Furthermore, METTL1/WDR4 complex can increase miRNA expression by boosting the processing and maturation of pri-miRNA, down-regulating the downstream oncogenes [224, 236]. In summary, METTL1/WDR4 complex serves an oncogenic role via m 7G modifying tRNA, while its effect on miRNA seems tumor suppressive. The function of m 7G writers on other RNAs remains to be explored.

### The role of RNA modification in cancer metabolism

#### The RNA modification and glucose metabolism

**Fig. 5:**
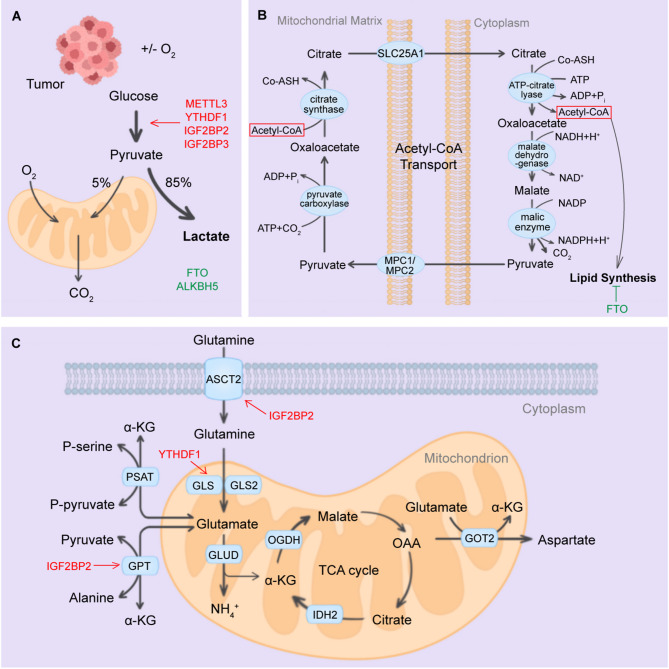
Roles of RNA modification regulators in cancer metabolism. (**A**)The RNA regulators METTL3, YTHDF1, IGF2BP2, and IGF2BP3 can enhance glycolysis in cancers, while the erasers FTO and ALKBH5 act as suppressors in this process. (**B**) FTO can inhibit lipid synthesis. (**C**) The main process of glutamine metabolism. IGF2BP2 can target ASCT2 and GPT, facilitating glutamine metabolism. YTHDF1 can promote protein synthesis of GLS, regulating glutamine metabolism

Glucose metabolism is characterized by anaerobic glycolysis, aerobic respiration, the pentose phosphate pathway, and glycogen metabolism. Metabolic reprogramming is a hallmark of cancer cells, with aberrant glucose metabolism being a significant component. Highly invasive cancer cells tend to have increased glycolytic activity and lactic acid fermentation, namely the Warburg effect (Fig. 5).

HK2 is the first crucial rate-limiting enzyme in glycolysis. METTL3 can target the 3’-UTR of HK2 mRNA and recruit YTHDF1 to enhance the stability of HK2, thereby facilitating the Warburg effect in cervical cancer [237]. Moreover, METTL3 can directly bind to the 5’-UTR or 3’-UTR of HK2 and the 3’-UTR of glucose transporter(GLUT1), stabilizing gene expression via IGF2BP2 or IGF2BP3 and activating the glycolysis pathway to modulate tumor cell progression [238]. The erasers, such as ALKBH5 and FTO, also act as crucial regulators in glycolysis. ALKBH5 can suppress cell proliferation and enhance cisplatin sensitivity in bladder cancer through m6 A-CK2α-dependent glycolysis regulation [239]. FTO can inhibit the expression of apolipoprotein E (APOE) and repress the glycolysis and cancer growth in an m6 A-dependent manner [240]. Moreover, the expression of FTO is significantly down-regulated in papillary thyroid cancer (PTC).

### The RNA modification and fatty acid metabolism

Fatty acid oxidation is an essential source of cellular energy, and tumor cells present enhanced adipogenesis because of the high demand for nutrients used in their survival, proliferation, invasion, and metastasis [241]. In clear cell renal cell carcinoma (ccRCC), oxoglutarate dehydrogenase-like (OGDHL) is notably down-regulated, leading to the inhibition of lipid synthesis [242]. Furthermore, FTO-mediated OGDHL m 6A demethylation represses its expression in ccRCC and regulates lipid metabolism, promoting tumor progression through the FTO/OGDHL/TFAP2A/FASN axis.

### The RNA modification and amino acid metabolism

The amino acid metabolism in the body primarily involves two parts: ①the synthesis of peptides or proteins, ②energy release through deamination, transamination, and TCA cycle oxidation. Cancer cells usually elevate the glutamatelyase to maintain the functional TCA cycle, and glutamine decomposition is an important hallmark of tumor energy metabolism as well [87]. The m 6A reader IGF2BP2 facilitates AML proliferation by modulating the expression of critical targets, including GPT2, MYC, and SLC1A5 in the glutamine metabolism pathways [243] (Fig. 5). Besides, YTHDF1 is significantly up-regulated in CRC. Targeting YTHDF1 can regulate glutaminase (GLS)-mediated glutamine metabolism, re-sensitizing cisplatin-resistant colon cancer cells [244]. These studies elucidate the key role of RNA modification in cancer metabolism and suggest the possibility of targeting RNA modification pathways to enhance the efficacy of cancer therapy.

### Applications of RNA modifications in cancer diagnosis, prognosis, and treatment

#### RNA modification regulators are potential targets for cancer therapy

**Fig. 6:**
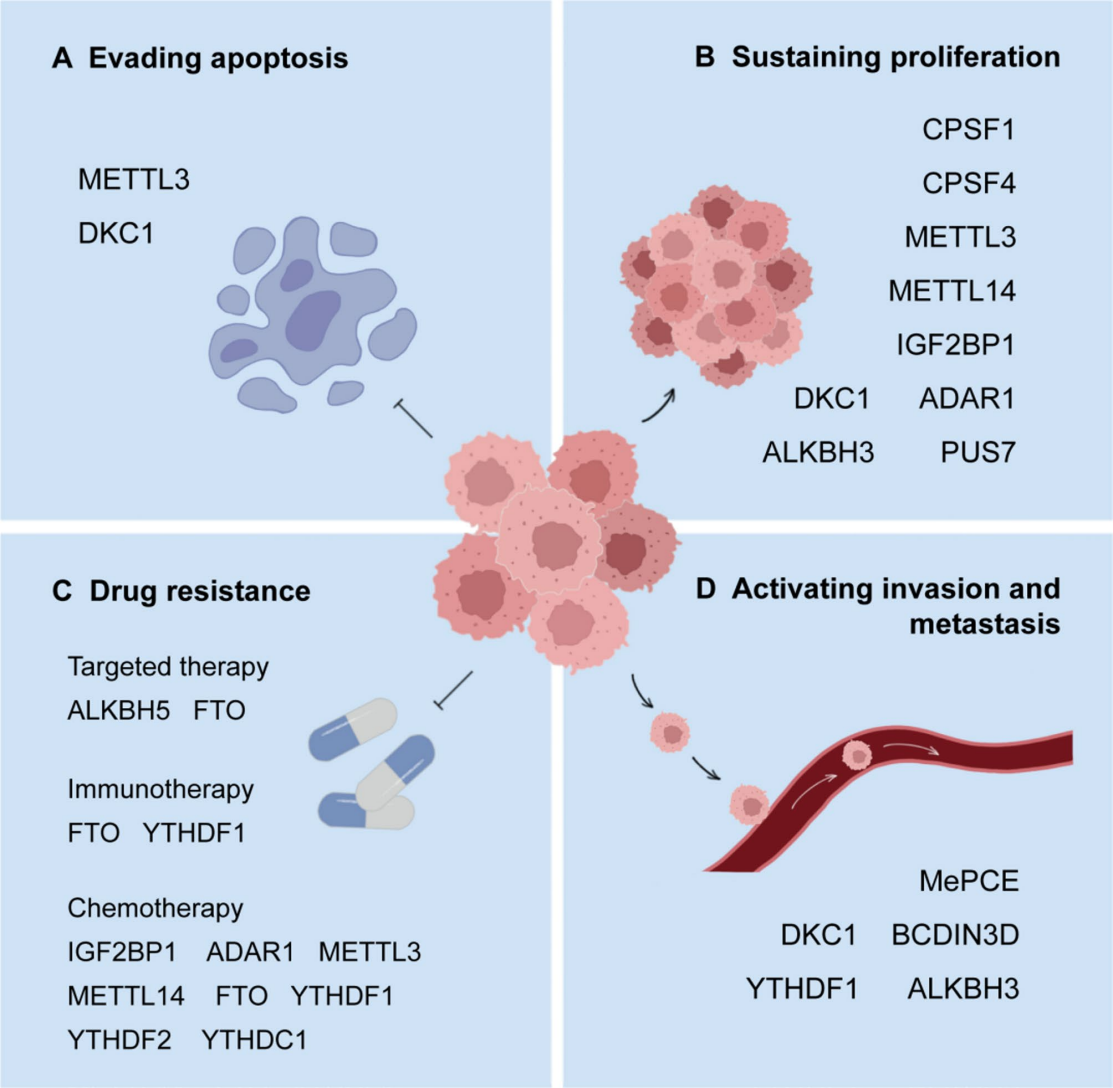
Roles of RNA modification regulators in tumor progression. (**A**) METTL3 plays an oncogenic role by methylating the BCL-2, PTEN, and c-MYC mRNAs, facilitating their translation, leading to the inhibition of apoptosis in AML. In NSCLC, DKC1 synergizes with PCAT1 to stimulate cell proliferation and invasion as well as inhibit apoptosis. (**B**) The up-regulation of CPSF1 accelerates cell proliferation in HCC. CPSF4 can stimulate the growth and progression of colon cancer and lung cancer. The METTL3-METTL14 complex enhances AML proliferation by up-regulating oncogenic genes. In CRC, IGF2BP1 promotes tumor proliferation by reducing the expression of epithelial markers. In NSCLC, DKC1 synergizes with PCAT1 to stimulate cell proliferation and invasion as well as inhibit apoptosis. ADAR1 can mediate A-to-I editing of miR-25-3p and miR-125a-3p binding sites, inducing cell proliferation and drug resistance in breast cancer. ALKBH3 promotes the proliferation of cancer cells via demethylating tRNAs and generating tRNA-derived small RNAs. PUS7 can enhance cell proliferation in glioblastoma. (**C**) ALKBH5-mediated m6 A demethylation of GLUT4 mRNA promotes drug resistance to HER2-targeted therapy. FTO can promote resistance to targeted therapy, immunotherapy, and chemotherapy. YTHDF1 can increase p65 translation and promote anti-PD-1 resistance. IGF2BP1, METTL3, METTL14, YTHDF1, YTHDF2, and YTHDC1 can promote chemotherapy resistance. (**D**) MePCE stabilizes 7SK and provides invasive potential to breast cancer cells. BCDIN3D attenuates the expression of miRNAs and promotes the invasive potential of breast cancer cells. YTHDF1 can enhance the translation of EIF3C, leading to metastasis of ovarian cancer. The demethylation of m 1A by ALKBH3 increases the stability of CSF-1 mRNA, promoting the invasion of breast cancer and ovarian cancer

RNA-modifying enzymes are proven to play vital roles in the occurrence, prognosis, and progression of cancers, indicating their great potential in anti-tumor therapy(Fig. 6). The reasonable strategy to generate new therapeutic agents includes traditional medicine-related techniques and modern drug-developing approaches based on AI technology and chemosynthesis.

Particularly, natural compounds can be used as a biochemical library for targeted anti-tumor drug discovery. A previous study screened several inhibitors of FTO, a human RNA demethylase mentioned above, and found the best inhibitor rhein [245]. The rhein can reversibly combine with FTO and competitively inhibit the recognition of m6 A substrates. Given the oncogenic role of FTO in many cancer types, rhein may act as a potential lead for new treatment. In addition, sulforaphane can diminish m6 A modification levels, thus inducing cell cycle arrest, apoptosis, and autophagy in breast cancer [246]. Saikosaponin-d (SsD) can suppress cell proliferation and stimulate apoptosis in AML by directly targeting FTO/m^6^A signaling and inhibiting downstream pathways [247]. Conventional drugs, such as meclofenamic acid 2 (MA2), were also identified as a modulator of RNA modification. MA2 is a nonsteroidal anti-inflammatory drug (NSAID) approved by the FDA and can selectively inhibit demethylation mediated by FTO [248]suppressing glioblastoma stem cell-initiated tumor growth [108].

In addition to exploring the new use of traditional drugs, structure-guided drug design based on virtual screening, computational docking, and modern chemosynthesis is widely applied in the field of RNA enzyme drug development. A high-throughput screen identified STM2457 as a highly selective and potent catalytic inhibitor of METTL3 [249]. STM2457 can reduce AML growth and increase differentiation and apoptosis by decreasing the m 6A level on leukemogenic mRNAs and the expression of these mRNAs. Other small molecule inhibitors that target METTL3/METTL14 were tested in different cancer cell lines [250]. STC-15, an orally bioavailable small molecule, is currently undergoing a multi-center phase I clinical trial to evaluate the safety in advanced tumors (NCT05584111). FTO is also recognized as an anti-tumor target with great promise. The FTO inhibitor R-2-hydroxyglutarate (R-2HG) displays anti-tumor activity in non-IDH mutant AML and glioma by targeting FTO/m^6^A/MYC/CEBPA signaling [110]. Besides, FB23 and FB23-2 are two potential FTO inhibitors that can directly bind to FTO and suppress the m6 A demethylase activity of FTO [251]. FB23-2, a new derivative of MA, can inhibit the proliferation and stimulate differentiation and apoptosis of AML cells. Compared to FB23-2, CS1 and CS2 bind to the catalytic core of FTO, repressing its activity in AML more efficiently. Moreover, CS1 and CS2 can repress immune escape via targeting leukocyte immunoglobulin-like receptor subfamily B4 (LILRB4), which is an immune checkpoint protein in AML cell lines [252]. A recent study presented the antiproliferative effects of oxetane-based compounds in the proliferation of AML, glioblastoma, and gastric cancer [253]. As a member of oxetane-based compounds, FTO-43 significantly inhibits FTO and shows potency comparable to 5-fluorouracil (5’FU). Furthermore, thiram can suppress the interaction between TRMT6 and TRMT61A, inhibiting HCC growth [141]. An epigenetic cancer therapeutic agent, azacytidine, has been developed as an epigenetic cancer therapeutic agent due to its ability to inhibit RNA methylation of the major substrate of RNA methyltransferase DNMT2 in human cancer cell lines [254].

Photoactivated compounds are constructed to modulate RNA modification as well. Lan et al. developed a photo-caging substituent-linked MPCH which activated METTL3/14 and raised cellular m 6A levels after short UV light irradiation [255]. This gain-of-function was also observed in live human cancer cells, and the cytotoxicity caused by ultraviolet radiation was reduced to a minimum. The photo-caging strategy might be a potential technique to precisely regulate RNA modification and tumor development.

### RNA modifications are effective biomarkers for cancer treatment efficacy

#### Chemotherapy

RNA modification is related to the drug resistance of cisplatin, anthracyclines, 5-fluorouracil (5-FU), and gemcitabine in tumors. The ‘writer’ METTL3 can stabilize mRNA of factor-activating enhancer-binding protein 2 C (TFAP2C) and activate DNA repair gene BRCA1, thus making seminoma insensitive to cisplatin [256]. Additionally, METTL3 can boost the maturation of pri-microRNA-221-3p, facilitating adriamycin(ADR; also named doxorubicin) resistance in breast cancer cells [257]. METTL14 plays a contrary role in different cancers. For instance, the low expression of METTL14 in CRC enhances the stability of pri-miR-17 mRNA and increases the expression of miR-17-5p in an m6 A-dependent manner, inducing chemotherapy resistance to 5-FU [258]. Conversely, METTL14 is overexpressed in gemcitabine-resistant pancreatic cancer cells and mediates the up-regulation of cytidine deaminase, promoting gemcitabine resistance in pancreatic cancer [259].

The ‘erasers’ contribute to chemotherapy drug resistance as well. The ALKBH5-HOXA10 loop can mediate JAK2 m6 A demethylation and activate JAK2/STAT3 signaling pathway, facilitating cisplatin resistance in epithelial ovarian cancer (EOC) [260]. In contrast, ALKBH5 can sensitize bladder cancer cells to cisplatin by m 6A-casein kinase 2 (CK2)α-mediated glycolysis [261]. ALKBH5 also plays a chemo-sensitizing role by reducing the m 6A level of Wnt inhibitory factor 1 (WIF-1) and repressing the activation of the Wnt signaling pathway [262]. The overexpression of ALKBH5 can sensitize PDAC cells to gemcitabine. Moreover, FTO promotes the doxorubicin resistance driven by signal transducer and activator of transcription 3 (STAT3) in breast cancer [263]. Inhibitors targeting FTO or STAT3 can reduce doxorubicin resistance, which may be a potential therapeutic strategy for breast cancer.

Recent studies suggest RNA modification readers are important regulators in drug resistance as well. YTHDF1 mediates cisplatin resistance through glutaminase 1 (GLS1)- glutamine metabolism axis in CRC [244]. YTHDF2 facilitates cyclin-dependent kinase inhibitor 1B (CDKN1B) mRNA degradation and plays a cisplatin-desensitizing role in intrahepatic cholangiocarcinoma (ICC) [264]. Furthermore, YTHDC1 can bind to m6 A-modified EGF mRNA and promote EGF synthesis, thus enhancing homologous recombination repair(HR) during chemotherapy and leading to adriamycin resistance in breast cancer cells [265].

The development of inhibitors against the above RNA modification regulators or targeting their pathways combined with chemotherapy is a potential cancer treatment strategy. Li et al. found that METTL3 mediated chemotherapy resistance in patients with AML recurrence while METTL3 inhibitor STM2457 reversed drug resistance of chemo-resistant AML cells [266]. Additionally, previous research reported that leukemia patients with isocitrate dehydrogenase (IDH, an enzyme that could produce R-2HG) mutation were more sensitive to chemotherapy [267]. Su et al. further revealed the synergistic effect between FTO inhibitor R-2HG and traditional chemotherapy drugs such as azacytidine, daunorubicin, and decitabine [110].

### Radiotherapy

The resistance of tumor cells to radiotherapy involves multiple genes and various mechanisms, such as DNA double strand breaks (DSB) repair [268]. Particularly, METTTL3 plays a vital role in the radio-resistance of glioma stem-like cells (GSCs) in an m6 A-dependent manner [269]. Silencing METTL3 can decrease the DSB repair and enhance the sensitivity to γ-irradiation in GSCs. The m 6A RNA demethylase ALKBH5 shows opposite effects in different cancer species. ALKBH5 is overexpressed in glioblastoma stem cells (GBMSCs) and promotes their radio-resistance by modulating homologous recombination (HR) [270]. However, ALKBH5 mediates the demethylation of circAFF2, which can facilitate the interaction of CAND1 with Cullin1, thus enhancing the radio-sensitivity of CRC cells [271]. The m6A reader YTHDC2 is up-regulated in radio-resistant nasopharyngeal carcinoma (NPC) cells [272]. YTHDC2 can bind to insulin-like growth factor 1 receptor (IGF1R) mRNA and activate IGF1R-AKT/S6 signaling, promoting radio-resistance of NPC cells (See Table 2).

**Table 2:** The function of RNA modification regulators in therapy resistance

Therapy type	Regulator	Drug	Cancer	Regulation	Target/Axis
Chemotherapy	METTL3	Cisplatin	Seminoma	Promote resistance	TFAP2C
		Adriamycin	Breast cancer	Promote resistance	Pri-microRNA-221-3p
	METTL14	5-FU	CRC	Enhance sensitivity	Pri-miR-17
		Gemcitabine	Pancreatic cancer	Promote resistance	Cytidine deaminase
	ALKBH5	Cisplatin	EOC	Promote resistance	JAK2/STAT3 signaling
		Cisplatin	Bladder cancer	Enhance sensitivity	CK2α-mediated glycolysis
		Gemcitabine	PDAC	Enhance sensitivity	WIF-1
	FTO	Adriamycin	Breast cancer	Promote resistance	STAT3
	YTHDF1	Cisplatin	CRC	Promote resistance	GLS-glutamine metabolism axis
	YTHDF2	Cisplatin	ICC	Promote resistance	CDKN1B
	YTHDC1	Adriamycin	Breast cancer	Promote resistance	EGF
Radiotherapy	METTL3	-	Glioma	Promote resistance	GSC/DSB repair
	ALKBH5	-	Glioma	Promote resistance	GBMSC/HR
		-	CRC	Enhance sensitivity	ALKBH5/YTHDF2/circAFF2/Cullin-NEDD8 axis
	YTHDC2	-	NPC	Promote resistance	IGF1R/AKT/S6 signaling axis
Targeted therapy	METTL3	Anlotinib	OSCC	Promote resistance	FGFR3
		Sorafenib	HCC	Enhance sensitivity	FOXO3-mediated autophagy
	METTL1/ WDR4	Lenvatinib	HCC	Promote resistance	EGFR signaling pathway
	FTO	TKIs	-	Promote resistance	-
Immunotherapy	METTL3	Anti-PD-L1 monoclonal antibody	Bladder cancer	Promote resistance	JNK signaling
	METTL3/14	Anti-PD-1 monoclonal antibody	CRC	Promote resistance	IFN-γ-Stat1-Irf1 signaling
	FTO	Anti-PD-1 monoclonal antibody	Melanoma	Promote resistance	PD-1/CXCR4/SOX10
	ALKBH5	Anti-PD-1 monoclonal antibody	Melanoma	Promote resistance	Mct4/Slc16a3
	IGF2BP3	Oncolytic herpes virotherapy	Glioma	Promote resistance	IGF2BP3/MIB1/FTO axis

Note: CRC, colorectal cancer; EOC, epithelial ovarian cancer; PDAC, pancreatic ductal adenocarcinoma; ICC, intrahepatic cholangiocarcinoma; GSC, glioma stem-like cells; DSB, double strand breaks; GBMSC, glioblastoma stem cells; HR, homologous recombination; NPC, nasopharyngeal carcinoma; OSCC, oral squamous cell carcinoma; HCC, hepatocellular carcinoma

### Targeted therapy

Targeted therapy represented by tyrosine kinase inhibitors (TKI) has shown great efficacy in lung cancer and leukemia while drug resistance and recurrence affect the curative effect. METTL3 can modify fibroblast growth factor receptor (FGFR3, an anlotinib target) m6 A methylation in oral squamous cell carcinoma (OSCC), and the knockdown of METTL3 enhances anlotinib sensitivity of OSCC cells [273]. METTL3 can also improve the stability of FOXO3 in a YTHDF1-dependent manner, thus regulating sorafenib resistance in HCC [274]. METTL1/WDR4-mediated m 7G modification on tRNA promotes lenvatinib resistance in HCC [275]. In addition, Yan et al. have found that cells with FTO up-regulation are more tolerant to TKI, whereas FTO inactivation makes resistant cells re-sensitive to TKI [276].

### Immunotherapy

The development of immunotherapy has ushered in a new era of tumor treatment and demonstrates encouraging therapeutic potential [277]. For instance, immune checkpoint inhibitors(ICIs) have shown strong anti-tumor activity in the treatment of multiple tumors, such as melanoma, as well as other solid tumors, and a number of tumor immunotherapy drugs have been approved for clinical application. Nevertheless, a large proportion of patients do not benefit from treatment due to drug resistance. RNA modification plays a critical role in tumor immunity, including modulating various immune cells directly, regulating the expression of immune checkpoints, and inducing metabolic reprogramming [278].

Recent studies have elucidated the effect of RNA modification on tumor immunotherapy efficacy. JNK signaling pathway regulates m6 A abundance on PD-L1 mRNA and affects PD-L1 expression, contributing to immune escape in bladder cancer [279]. Administration of JNK inhibitors may be a potential strategy to enhance the efficacy of immunotherapy for bladder cancer. Besides, the depletion of METTL3 and METTL14 enhances the response of CRC and melanoma to anti-PD-1 therapy through promoting IFN-γ-Stat1-Irf1 signaling [280].

As for erasers, FTO can promote anti-PD-1 resistance by modulating m6 A methylation in several vital protumorigenic melanoma cell-intrinsic genes, PD-1, SOX10, and CXCR4 [109]. Hence, combining FTO inhibitors with anti-PD-1 monoclonal antibodies may resensitize melanoma to immunotherapy. ALKBH5 regulates the expression of Mct4/Slc16a3 and affects the abundance of myeloid-derived suppressor cells (MDSC) and Treg in the tumor microenvironment(TME) [281]. The application of ALKBH5 inhibitors enhances the efficacy of anti-PD-1 therapy in melanoma.

The oncolytic virus is another research hotspot in the field of immunotherapy. Lately, Dai et al. reported that the up-regulation of IGF2BP3 led to increased neutrophil extracellular traps (NETs), which influenced the curative effect of oncolytic virotherapy [282]. BET inhibitors could enhance the oncolytic activity of oncolytic herpes simplex virus(oHSV) by preventing IGF2BP3-induced NETosis in malignant glioma. The above researches suggest RNA modification regulators as potential therapeutic targets to enhance the outcome of chemotherapy, radiotherapy, targeted therapy, and immunotherapy in multiple cancers.

### RNA modifications are potential biomarkers for cancer diagnosis and prognosis

RNA modification expression profile can act as a potential clinical prediction model. A recent study established an RNA modification ‘writer’ Score (WM_Score) model, which could indicate RNA modification patterns and prognosis of CRC patients [20]. Importantly, this model contributed to the selection of CRC drugs and the prediction of the therapeutic efficacy of PD-L1 blockade. Moreover, RNA epigenetic marks are potential biomarkers in cancers. A prospective analysis verified that Ψ served as a potential risk factor for ovarian cancer [283].

### Application of RNA modification in mRNA vaccines

#### The role of mRNA vaccines in cancer therapy

Cancer vaccines, including oncolytic virus (OV) vaccines, peptide vaccines, cell vaccines, and nucleic acid vaccines, mainly activate the human immune system to resist cancer cells and have been proven to be effective in some cancer types [284–288]. However, OVs are natural or genetically edited live viruses, so dose changes are difficult to control, and the virus can cause the host antiviral immune response [289]. Cell vaccines mainly include dendritic cell (DC) therapy and chimeric antigen receptor T cells (CAR-T) therapy. Most DC therapies require the isolation of DCs from the patient, which is time-consuming. CAR-T therapy has some defects, such as autotoxicity, off-target effects, and immunosuppression, so it has not been applied to solid tumors [290–292]. Peptide vaccines lack clinical use because they are difficult to synthesize in vitro and deliver in vivo.

The mRNA vaccines have emerged as a promising new strategy for cancer immunotherapy. Saint et al. first reported the clinical trial of a multi-antigen mRNA vaccine in melanoma patients in 2017 [293]. The effectiveness of COVID-19 mRNA vaccines has further made mRNA vaccines a research hotspot. Unlike traditional vaccines, mRNA vaccines express tumor antigens in antigen-presenting cells(APCs) by introducing antigen-encoding mRNA rather than the antigen into cells, thereby stimulating subsequent immune responses [294]. Compared with peptide vaccines, mRNA vaccines are easier to synthesize in vitro. Moreover, mRNA vaccines eliminate the potential risk of nuclear integration in contrast to DNA vaccines [295]. mRNA vaccines have the advantages of high efficiency, strong safety, and easy large-scale production, while their application is still limited by innate immunogenicity and instability. Appropriate RNA modification can improve the above defects.

#### Cap and tail RNA modifications modulate the stability of the mRNA vaccine

**Fig. 7:**
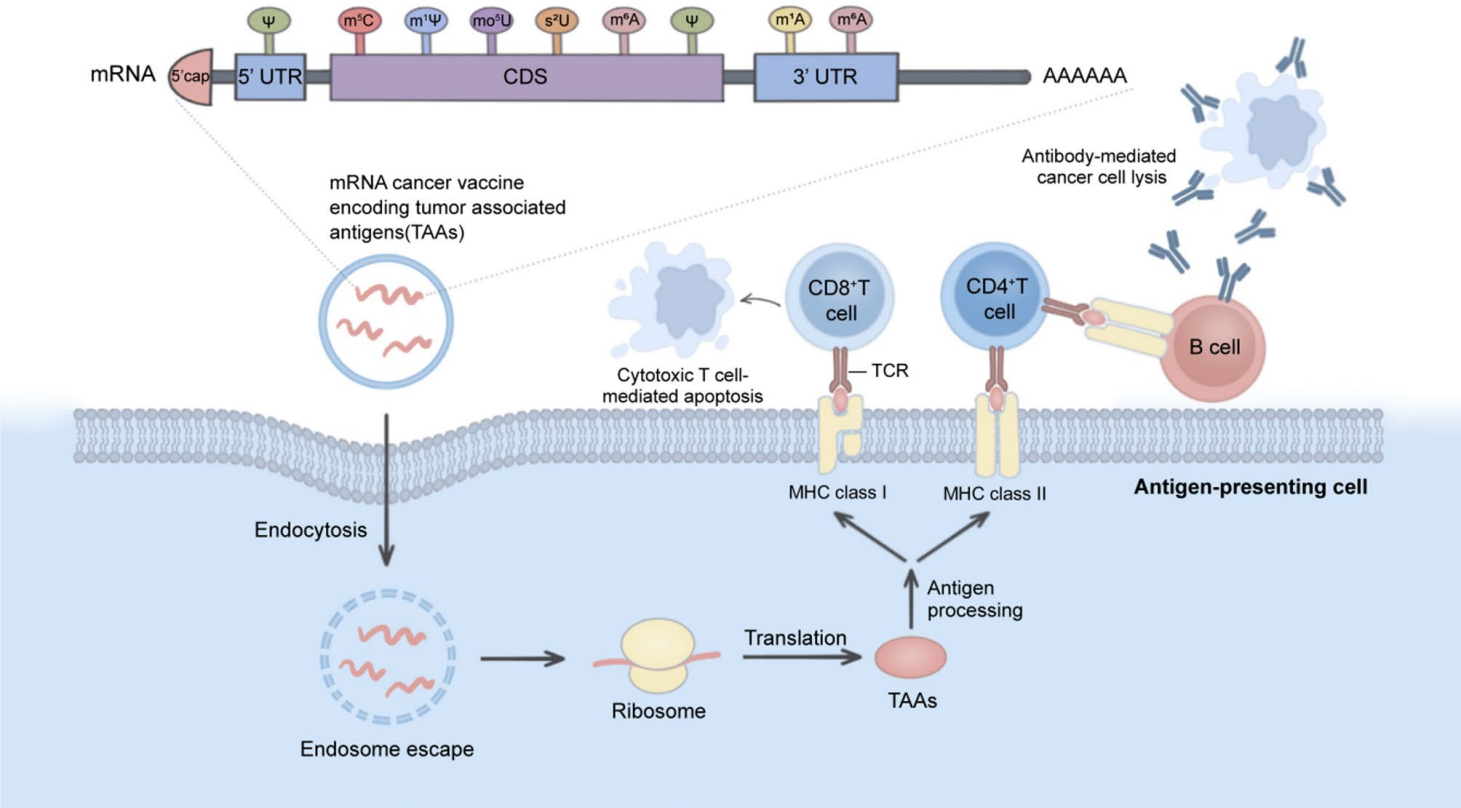
Design of mRNA cancer vaccines and nucleotide modifications. The mRNAs synthesized in vitro have five core components: 5’ cap, 5’ UTR, CDS, 3’ UTR, and poly(A) tail. Appropriate RNA modification is necessary to enhance mRNA stability and reduce innate immunogenicity. mRNA vaccine which encodes TAAs undergoes endocytosis into antigen-presenting cells (APCs). Whereafter, the mRNA is released into the cytoplasm of APCs and translated into TAAs by ribosomes. The processed TAAs interact with major histocompatibility complex (MHC) class I and are presented to the cell surface, thereby activating CD8^+^ T cells and leading to the apoptosis of cancer cells. The processed TAAs can also interact with MHC class II, activating CD4^+^ T cells and inducing B cells to generate antibodies to mediate tumor cell killing

The mRNA synthesized in vitro is extremely unstable and is easily inactivated after entering the body, which is one of the main factors limiting the application of mRNA vaccines in cancer therapy. To enhance mRNA stability and improve translation efficiency, exogenous RNA used for treatment should preferably have a 5’cap and poly(A) tail to mimic the endogenous mRNA in the cytoplasm as closely as possible. In sum, the mRNAs synthesized through the in vitro transcription (IVT) strategy are supposed to have five core components: 5’ cap, 5’ UTR, coding sequence (CDS), 3’ UTR, and poly(A) tail (Fig. 7).

The m7 G-modified 5’ cap of eukaryotic mRNA serves as a protective structure for mRNA, boosts promoter binding, and enhances the efficiency of protein translation [296]. Exogenous mRNA produced by IVT can synthesize 5’cap-like structures and capsid enzymes using vaccinia virus capping enzyme (VCE) or bacteriophage polymerases [297, 298]thus improving mRNA stability. The addition of poly(A) tails contributes to mRNA stability as well. The poly (A) tail region contains many modification sites. Previous research has found that guanylation can protect mRNA from rapid deadenylation [299]. In contrast, uridylation of the poly (A) tail can facilitate mRNA decay [300].

Furthermore, adjusting the sequence in 5’UTR and 3’UTR regions, such as adding the Kozak sequence after the 5’UTR and replacing the adenylate-uridylic acid-rich sequence of the 3’UTR, can improve mRNA translation efficiency and inhibit mRNA degradation, respectively [301, 302]. Various RNA modifications in the 5’UTR region of endogenous RNA, including m 1A, m6 A, m5 C, Ψ, and N6,2’-O-dimethyladenosine (m6 Am) [303, 304]and the modifications in the 3’UTR region, such as m 6A, m5 C, and Ψ, also provide a vital reference model for the producing of mRNA vaccines in vitro [196, 304, 305].

#### Internal RNA modifications reduce the immunogenicity of mRNA vaccines

Exogenous RNA mainly comes from pathogens such as bacteria and viruses which invade the human body. These RNAs can be recognized by pattern recognition receptors (PRRS) and activate the innate immune response [306]. As endogenous RNA usually contains many modifications, unmodified mRNA vaccines can be easily recognized as ‘foreign invaders’ and eliminated by the immune system.

RNA modifications can substantially reduce innate immunogenicity and improve mRNA vaccine efficacy. For instance, modified mRNA containing Ψ, m5 C, m6 A, N1-methylpseudouridine (m1 Ψ), 5-methoxyuridine (mo 5U), or 2-thiouracil (s2 U) present reduced immunogenicity [307, 308]. Specifically, U is the key factor in mRNA that mediates unwanted immune responses, so the replacement of U with Ψ allows mRNA to escape immune surveillance [307]. The m5 C or m1Ψ modified mRNA has an enhanced ability to prevent TLR3 activation as well as downstream immune signaling [309]. Inosine evades immune signaling via PRR MDA5 [310]. Besides, several ribose methylations can repress cytokine production which is downstream of PRR TLR7 [311, 312]. Of note, currently marketed mRNA vaccines for COVID-19 have already used N1-methyl pseudouridine triphosphate (m 1ΨTP) instead of uridine triphosphate (UTP) to enhance efficacy.

In summary, the optimization of mRNA structure can gradually improve the defects of mRNA vaccines. Besides, upgrading vaccine delivery systems is also critical [313]. In the clinical scenario, using mRNA vaccine therapy alone may be effective in early-stage cancer while in advanced cancer combination therapy should be considered (NCT03897881, NCT04534205). The development of personalized and preventive mRNA vaccines has a broad prospect in the field of tumor prevention and treatment.

#### Conclusion and perspective

**Fig. 8:**
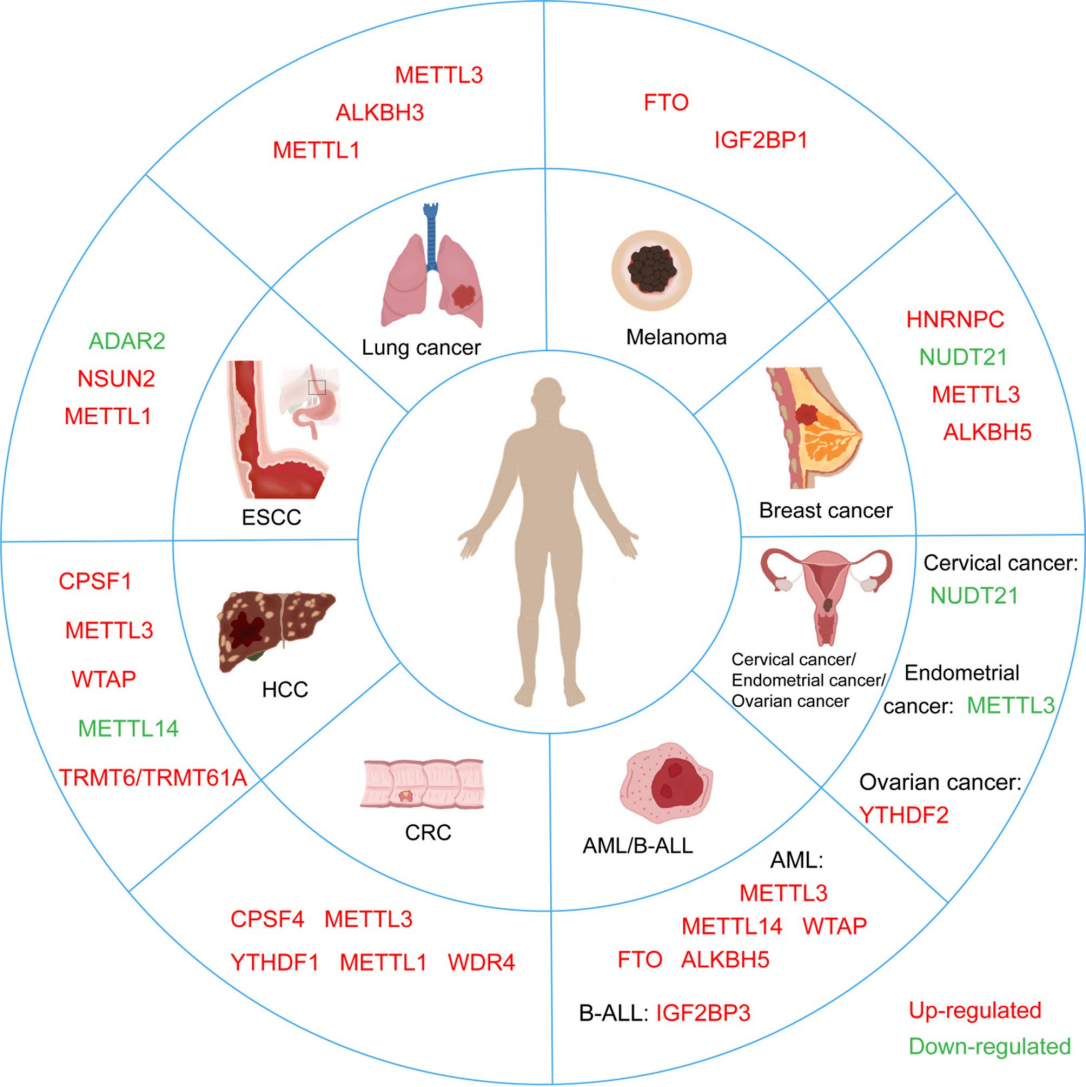
Dysregulation of RNA modification regulators in human cancers. The up-regulated RNA modification regulators in lung cancer, esophageal squamous cell carcinoma(ESCC), hepatocellular carcinoma(HCC), colorectal cancer(CRC), acute myeloid leukemia(AML), cervical cancer, endometrial cancer, ovarian cancer, breast cancer, and melanoma are highlighted in red, while the down-regulated RNA modification regulators are highlighted in green

The diverse epigenetic modifications of RNA reveal their critical role in post-transcriptional gene regulation. In this review, we summed up nine kinds of modifications that occurred on the cap, tail, and internal section of RNAs, and investigated their effects on cancer development. These RNA modifications can regulate the growth, proliferation, metastasis, apoptosis, and metabolism of cancer cells by affecting RNA biological functions. Three classes of RNA modification regulators, writers, erasers, and readers, are often abnormally expressed in tumors, serving as oncogenic factors or tumor suppressors (Fig. 8).

Particularly, the writers of many modifications have been studied in many cancers, while the erasers and readers for certain RNA modifications, such as A-to-I editing, m 7G, and Ψ, have not been identified yet. We discussed the aberrant up-regulation or down-regulation of RNA-modifying enzymes as well as their influence on the hallmarks of cancer [314]. However, an enzyme can play the opposite roles in different types of cancer, which may be relevant to the variety of RNA modifications and substrate RNA species and the existence of tumor heterogeneity. Furthermore, published research mainly focused on the roles of RNA modifications on downstream pathways and their mechanism, whereas few studies ascertained the upstream reactions that led to the abnormal abundance of RNA epigenetic marks. Although numerous articles reported that RNA modifications could affect most hallmarks of cancer, the limited knowledge of RNA modifications’ effects on angiogenesis, immune escape, and metabolism calls for further exploration.

Along with the progression of mechanistic research, the field of RNA-modifying enzyme drug development has gained more attention. Targeting RNA modification regulators is a potential therapeutic strategy because RNA modification is critically related to tumor progression, and the structures of most regulators are available. Natural products and traditional medicine can be used as a biochemical library to screen modulators of RNA enzymes. Over 60% of anti-tumor drugs are of natural origin or include pharmacophores of natural compounds [315]. The safety and efficiency of traditional drugs have been verified by plenty of clinical trials, so traditional medicines are reliable sources for screening novel therapeutic agents. The combination of natural agent database and AI-assisted techniques, such as molecular docking simulation and quantitative structure-activity relationship models, might be a promising drug discovery approach [19].

The development of clinical potential of RNA modification is inseparable from the progress of epitranscriptomics. Increasingly improved methods for the detection of RNA modifications have enabled in-depth mechanistic exploration of the epitranscriptome. For example, long-read nanopore sequencing may be able to detect RNA modifications at isoform resolution [316]. Single-cell and spatial RNA sequencing (RNA-seq) have enabled the construction of more detailed and dynamic transcriptomic maps, expanding our understanding of the temporal, spatial, and mechanistic dimensions governing RNA function and expression [317].

While the clinical potential of RNA modifications is evident, current methodologies face critical limitations. For instance, detecting low-abundance modifications relies on antibody-based techniques, which suffer from antibody cross-reactivity and limited resolution. Moreover, the dynamic nature of modifications poses challenges in capturing transient epitranscriptomic changes during cancer progression. In the era of paradigm shifts driven by big data and artificial intelligence (AI), accessible mapping of RNA modifications should be combined with machine learning, deep learning, and large language models (LLMs) to drive the discovery of biomarkers for RNA modification-based diseases, especially cancer, to accelerate diagnosis and prognosis. And the expanding toolkit of RNA modification engineering will facilitate precise examination of the mechanisms by which RNA modifications regulate different biological processes [318].

Notably, the field of RNA-modifying enzyme drug design is still in its nascent stages. Although numerous modulators of RNA enzymes have been conceptualized, only a limited number have progressed to clinical trials for cancer treatment, primarily due to unresolved concerns regarding their safety and efficacy profiles. Surprisingly, high-throughput structure-based screening leads to an efficient, fast, and low-cost drug development strategy. It is anticipated that an array of new inhibitors and activators targeting RNA modifications will emerge, undergoing rigorous validation in subsequent clinical trials. The combination of these RNA modification drugs and representative cancer treatments, including chemotherapy, radiotherapy, targeted therapy, and immunotherapy, may present a potentially effective treatment strategy.

Besides, as a type of immunotherapy, mRNA vaccines have attracted wide attention due to their high efficacy, robust safety profile, and the feasibility of large-scale production. The design of IVT mRNA based on RNA modification technology helps to reduce the innate immunogenicity and enhance the stability and efficacy of the vaccine. Clinical trials for mRNA vaccines in cancer treatment are currently underway. It is imperative to tailor mRNA cancer vaccines to individual patients, and future research should focus on exploring the potential of combinatorial treatment strategies that incorporate mRNA cancer vaccines with other conventional therapeutic modalities.

## Data Availability

No datasets were generated or analysed during the current study.
